# Higher adiposity and mental health: causal inference using Mendelian randomization

**DOI:** 10.1093/hmg/ddab204

**Published:** 2021-07-16

**Authors:** Francesco Casanova, Jessica O’Loughlin, Susan Martin, Robin N Beaumont, Andrew R Wood, Edward R Watkins, Rachel M Freathy, Saskia P Hagenaars, Timothy M Frayling, Hanieh Yaghootkar, Jess Tyrrell

**Affiliations:** Genetics of Complex Traits, The College of Medicine and Health, RD&E Hospital, University of Exeter, Exeter EX2 5DW, UK; Genetics of Complex Traits, The College of Medicine and Health, RD&E Hospital, University of Exeter, Exeter EX2 5DW, UK; Genetics of Complex Traits, The College of Medicine and Health, RD&E Hospital, University of Exeter, Exeter EX2 5DW, UK; Genetics of Complex Traits, The College of Medicine and Health, RD&E Hospital, University of Exeter, Exeter EX2 5DW, UK; Genetics of Complex Traits, The College of Medicine and Health, RD&E Hospital, University of Exeter, Exeter EX2 5DW, UK; Mood Disorders Centre, School of Psychology, University of Exeter, Exeter, EX4 4QG, UK; Genetics of Complex Traits, The College of Medicine and Health, RD&E Hospital, University of Exeter, Exeter EX2 5DW, UK; Social, Genetic and Developmental Psychiatry Centre, Institute of Psychiatry, Psychology and Neuroscience, King's College London, London, SE5 8AF, UK; Genetics of Complex Traits, The College of Medicine and Health, RD&E Hospital, University of Exeter, Exeter EX2 5DW, UK; Genetics of Complex Traits, The College of Medicine and Health, RD&E Hospital, University of Exeter, Exeter EX2 5DW, UK; Genetics of Complex Traits, The College of Medicine and Health, RD&E Hospital, University of Exeter, Exeter EX2 5DW, UK

## Abstract

Higher adiposity is an established risk factor for psychiatric diseases including depression and anxiety. The associations between adiposity and depression may be explained by the metabolic consequences and/or by the psychosocial impact of higher adiposity. We performed one- and two- sample Mendelian randomization (MR) in up to 145 668 European participants from the UK Biobank to test for a causal effect of higher adiposity on 10 well-validated mental health and well-being outcomes derived using the Mental Health Questionnaire (MHQ). We used three sets of adiposity genetic instruments: (a) a set of 72 BMI genetic variants, (b) a set of 36 favourable adiposity variants and (c) a set of 38 unfavourable adiposity variants. We additionally tested causal relationships (1) in men and women separately, (2) in a subset of individuals not taking antidepressants and (3) in non-linear MR models. Two-sample MR provided evidence that a genetically determined one standard deviation (1-SD) higher BMI (4.6 kg/m^2^) was associated with higher odds of current depression [OR: 1.50, 95%CI: 1.15, 1.95] and lower well-being [ß: −0.15, 95%CI: −0.26, −0.04]. Findings were similar when using the metabolically favourable and unfavourable adiposity variants, with higher adiposity associated with higher odds of depression and lower well-being scores. Our study provides further evidence that higher BMI causes higher odds of depression and lowers well-being. Using genetics to separate out metabolic and psychosocial effects, our study suggests that in the absence of adverse metabolic effects higher adiposity remains causal to depression and lowers well-being.

## Introduction

Higher adiposity is an established risk factor for many psychiatric diseases including depression and anxiety. There is extensive evidence linking higher body mass index (BMI) to higher odds of depression (1) and anxiety (2,3) in the adult population, especially in women (4). Understanding the complex relationships between adiposity and mental health outcomes is crucial to facilitate public health and medical intervention planning. While several studies have attempted to test the directionality of associations between adiposity and mental health phenotypes including depression (5,6) and anxiety (3,7), determining causality in many studies is not trivial due to confounding or biases.

Mendelian randomization (MR) is a genetic approach that has provided some evidence that higher BMI (8–12) and higher body fat percentage (13) cause depression. To date, there are no studies using MR to specifically test the role of adiposity on anxiety. MR relies on the fact that genetic variation is randomly allocated at conception and assumes that genetic variants associated with the exposure (e.g. BMI) represent unconfounded proxies. The majority of studies to date have tested the role of adiposity on depression using summary statistic data from genome-wide association studies (GWAS) (10,11). These analyses are limited to the GWAS performed and leave several questions unanswered; for example, (a) does higher BMI cause depression in men and women separately? (b) are the relationships linear between BMI and depression? and (c) does antidepressant usage influence this relationship? Previous work by our group (12) attempted to address some of these questions in the UK Biobank but was limited by the mental health variables at the time.

The observational associations between obesity and depression or anxiety could be explained by (a) the physiological consequences of obesity, including higher inflammation (14,15) and/or (b) the psychological/social consequences of obesity. However, there is limited evidence about (a) whether adiposity is a causal risk factor for psychiatric diseases and (b) which component of higher adiposity (psychological/adverse social effect of excess weight, metabolic pathways or alternative pathways) causes the higher risk.

Here, we comprehensively test the relationship between higher BMI and well validated measures of depression and anxiety using data from the mental health questionnaire (MHQ) in up to 145 668 individuals of European ancestry in the UK Biobank. Firstly, we used three sets of genetic instruments in Mendelian randomization analyses: (a) 72 BMI genetic variants, (b) a set of 36 favourable adiposity variants that associate with higher adiposity, but a more favourable metabolic profile (characterized by lower triglycerides, higher HDL and lower type 2 diabetes risk) and (c) a set of 38 unfavourable adiposity variants that associate with higher adiposity and a less favourable metabolic profile (higher triglycerides, lower HDL and higher type 2 diabetes risk). We tested effects in men and women separately and explored non-linear relationships between BMI and mental health outcomes.

## Results

Table 1 summarizes the demographics of the 145 668 UK Biobank participants with valid genetic data, measured BMI and MHQ data available.

**Table 1:** Demographics of participants with mental health questionnaire data available

	All	Male	Female	*P* ^a^
*N*	145 668	63 462	82 206	
Age (SD)	56.6 (7.7)	57.2 (7.7)	56.1 (7.6)	<1.00E−15
BMI (SD)	26.8 (4.6)	27.3 (4.0)	26.4 (4.9)	<1.00E−15
TDI (SD)	−1.79 (2.8)	−1.83 (2.8)	−1.76 (2.8)	7.60E−14
Body fat % (SD)	30.8 (8.4)	24.5 (5.7)	35.6 (6.8)	<1.00E−15
Smoking status				<1.00E−15
Never (%)	83 335 (57.2)	33 275 (52.4)	50 060 (60.9)	
Former (%)	51 720 (35.5)	24 752 (39.0)	26 968 (32.8)	
Current (%)	9036 (6.2)	4590 (7.2)	4446 (5.4)	
Missing (%)	1577 (1.1)	845 (1.3)	732 (0.9)	
Major depression^b^ (%)	34 739 (23.9)	10 808 (17.4)	23 931 (29.1)	<1.00E−15
Severe major depression^c^ (%)	5483 (3.8)	1441 (2.3)	4042 (4.9)	<1.00E−15
Current depression^d^ (%)	2641 (1.8)	962 (1.5)	1679 (2.0)	6.70E−15
Severe current depression^e^ (%)	1787 (1.2)	662 (1.0)	1125 (1.4)	1.40E−08
Atypical depression^f^ (%)	2892 (2.0)	748 (1.2)	2144 (2.6)	<1.00E−15
GAD^g^ (%)	7218 (7.5)	2533 (5.5)	4685 (9.3)	<1.00E−15
Current GAD^h^ (%)	1844 (1.9)	646 (1.4)	1198 (2.4)	<1.00E−15
Mean CIDI severity (SD)	2.97 (3.0)	2.26 (2.8)	3.51 (3.0)	<1.00E−15
Mean PHQ9 severity (SD)	2.79 (3.7)	2.45 (3.5)	3.05 (3.8)	<1.00E−15
Mean GAD severity (SD)	2.15 (3.4)	1.75 (3.1)	2.45 (3.6)	<1.00E−15
Mean well-being score (SD)	12.7 (2.0)	12.7 (2.0)	12.7 (2.0)	1.60E−05

a Comparison males and females

b *N*_total_ = 145 583 *N*_male_ = 63 423 *N*_female_ = 82 160

c *N*_total_ = 145 583 *N*_male_ = 63 423 *N*_female_ = 82 160

d *N*_total_ = 145 667 *N*_male_ = 63 462 *N*_female_ = 82 205

e *N*_total_ = 145 667 *N*_male_ = 63 462 *N*_female_ = 82 205

f *N*_total_ = 145 583 *N*_male_ = 63 423 *N*_female_ = 82 160

g *N*_total_ = 96 658 *N*_male_ = 46 272 *N*_female_ = 50 386

h *N*_total_ = 96 642 *N*_male_ = 46 268 *N*_female_ = 50 374

### Higher BMI and body fat percentage are associated with adverse mental health outcomes in the UK Biobank

Observationally, higher BMI was associated with higher odds of depression and GAD (Table 2). For example, a 1-SD (4.6 kg/m^2^) higher BMI was associated with 1.16 [95%CI: 1.14, 1.17] higher odds of major depression, 1.56 [95%CI: 1.51, 1.62] higher odds of current depression and 1.10 [95%CI: 1.07, 1.13] higher odds of GAD. Following adjustment for GAD, higher odds of depression were still observed per 1-SD higher BMI, but the GAD findings were attenuated to the null when the model was adjusted for depression (Supplementary Material, Table S1). Higher BMI was also associated with lower well-being (Table 2).

**Table 2:** Observational associations between higher adiposity (using BMI and body fat percentage) and 10 mental health outcomes

		BMI			Body fat percentage		
**Mental health outcome**	**Strata**	***N* cases (controls)**	**OR (95% CI) per SD higher BMI**	***P*** ^a^	***N* cases (controls)**	**OR (95% CI) per SD higher body fat %**	***P*** ^**a**^
Major depression	All	34 739 (110844)	1.16 (1.14, 1.17)	<1.00E−15	34 338 (109526)	1.22 (1.20, 1.24)	<1.00E−15
	Males only	10 808 (52615)	1.16 (1.13, 1.19)	<1.00E−15	10 655 (51889)	1.22 (1.18, 1.26)	<1.00E−15
	Females only	23 931 (58229)	1.16 (1.14, 1.18)	<1.00E−15	23 683 (57637)	1.22 (1.19, 1.24)	<1.00E−15
Severe major depression	All	5483 (140100)	1.32 (1.28, 1.35)	<1.00E−15	5412 (138452)	1.43 (1.38, 1.48)	<1.00E−15
	Males only	1441 (61982)	1.50 (1.41, 1.60)	<1.00E−15	1416 (61128)	1.62 (1.50, 1.76)	<1.00E−15
	Females only	4042 (78118)	1.28 (1.24, 1.32)	<1.00E−15	3996 (77324)	1.38 (1.33, 1.44)	<1.00E−15
Current depression	All	2641 (143026)	1.56 (1.51, 1.62)	<1.00E−15	2601 (141347)	1.75 (1.66, 1.84)	<1.00E−15
	Males only	962 (62500)	1.67 (1.55, 1.80)	<1.00E−15	948 (61635)	1.78 (1.61, 1.96)	<1.00E−15
	Females only	1679 (80526)	1.53 (1.47, 1.60)	<1.00E−15	1653 (79712)	1.74 (1.63, 1.84)	<1.00E−15
Severe current depression	All	1787 (143880)	1.62 (1.55, 1.70)	<1.00E−15	1760 (142188)	1.83 (1.73, 1.95)	<1.00E−15
	Males only	662 (62800)	1.77 (1.62, 1.93)	<1.00E−15	653 (61930)	1.91 (1.70, 2.14)	<1.00E−15
	Females only	1125 (81080)	1.58 (1.49, 1.66)	<1.00E−15	1107 (80258)	1.81 (1.68, 1.94)	<1.00E−15
Atypical depression	All	2892 (142691)	2.15 (2.08, 2.23)	<1.00E−15	2860 (141004)	2.61 (2.48, 2.74)	<1.00E−15
	Males only	748 (62675)	2.47 (2.27, 2.69)	<1.00E−15	740 (61804)	2.87 (2.56, 3.22)	<1.00E−15
	Females only	2144 (80016)	2.09 (2.01, 2.18)	<1.00E−15	2120 (79200)	2.56 (2.42, 2.70)	<1.00E−15
Major depression without atypical depression	All	32 631 (110060)	1.13 (1.12, 1.15)	<1.00E−15	32 254 (108750)	1.19 (1.17, 1.20)	<1.00E−15
	Males only	10 294 (52381)	1.14 (1.12, 1.17)	<1.00E−15	10 147 (51657)	1.21 (1.17, 1.24)	<1.00E−15
	Females only	22 337 (57679)	1.13 (1.11, 1.15)	<1.00E−15	22 107 (57093)	1.18 (1.15, 1.20)	<1.00E−15
GAD	All	7218 (89440)	1.10 (1.07, 1.13)	4.60E−14	7132 (88403)	1.17 (1.13, 1.21)	<1.00E−15
	Males only	2533 (43739)	1.14 (1.09, 1.20)	9.80E−08	2501 (43143)	1.22 (1.15, 1.29)	1.70E−10
	Females only	4685 (45701)	1.09 (1.05, 1.12)	2.60E−08	4631 (45260)	1.15 (1.11, 1.20)	2.80E−12
Current GAD	All	1844 (94798)	1.17 (1.11, 1.22)	6.70E−11	1817 (93960)	1.22 (1.15, 1.30)	2.50E−10
	Males only	646 (45622)	1.31 (1.20, 1.44)	6.10E−09	635 (45000)	1.30 (1.16, 1.46)	1.20E−05
	Females only	1198 (49176)	1.12 (1.06, 1.18)	4.60E−05	1182 (48690)	1.19 (1.11, 1.28)	3.30E−06
**Mental health outcome**	**Strata**	***N* total**	***β* (95% CI) per SD higher BMI**	***P*** ^**a**^	***N* total**	***β* (95% CI) per SD higher body fat %**	***P*** ^**a**^
CIDI severity^b^	All	145 668	0.21 (0.20, 0.23)	<1.00E−15	143 949	0.27 (0.25, 0.29)	<1.00E−15
	Males only	63 462	0.19 (0.16, 0.21)	<1.00E−15	62 583	0.22 (0.19, 0.25)	<1.00E−15
	Females only	82 206	0.23 (0.21, 0.25)	<1.00E−15	81 366	0.30 (0.27, 0.33)	<1.00E−15
PHQ9 severity^b^	All	145 668	0.49 (0.47, 0.51)	<1.00E−15	143 949	0.61 (0.58, 0.63)	<1.00E−15
	Males only	63 462	0.45 (0.42, 0.49)	<1.00E−15	62 583	0.51 (0.47, 0.55)	<1.00E−15
	Females only	82 206	0.51 (0.48, 0.53)	<1.00E−15	81 366	0.67 (0.63, 0.70)	<1.00E−15
GAD severity^b^	All	145 069	0.11 (0.10, 0.13)	<1.00E−15	143 363	0.16 (0.13, 0.18)	<1.00E−15
	Males only	63 246	0.16 (0.14, 0.19)	<1.00E−15	62 371	0.20 (0.16, 0.23)	<1.00E−15
	Females only	81 823	0.09 (0.06, 0.11)	1.30E−13	80 992	0.13 (0.10, 0.16)	<1.00E−15
Well-being score^b^	All	141 447	−0.21 (−0.22, −0.20)	<1.00E−15	139 780	−0.27 (−0.28, −0.26)	<1.00E−15
	Males only	61 423	−0.18 (−0.20, −0.16)	<1.00E−15	60 570	−0.22 (−0.24, −0.20)	<1.00E−15
	Females only	80 024	−0.22 (−0.23, −0.21)	<1.00E−15	79 210	−0.30 (−0.32, −0.28)	<1.00E−15

a Adjusted for age, sex, centre, TDI and smoking status

b Severity scores used linear regression

Observationally, higher BFP was associated with higher odds of depression and GAD (Table 2). A 1-SD (8.4%) higher BFP associated with 1.22 [95%CI: 1.20, 1.24] higher odds of major depression, 1.75 [95%CI: 1.66, 1.84] higher odds of current depression and 1.17 [95%CI: 1.13, 1.21] higher odds of GAD. Higher BFP was associated with lower well-being, with a 1-SD higher BFP associated with lower well-being [ß: −0.27, 95%CI: −0.80, −0.26].

Observational associations were consistent when sex-stratified analyses were performed (Table 2). Adjusting the observational analyses for type 2 diabetes, alcohol intake, physical activity, hypertension, LDL, HDL, CVD and CAD slightly attenuated the effect estimates toward the null. However, higher BMI/BFP remained associated with higher odds of depression, anxiety and lower well-being although in females the confidence intervals for current GAD crossed the null (Supplementary Material, Table S2).

### Mendelian randomization analyses provided evidence that higher BMI causes depression and lowers well-being but is not associated with GAD

One-sample MR using unrelated individuals of European ancestry provided evidence for the causal role of higher BMI in depression. A genetically determined SD (4.6 kg/m^2^) higher BMI was associated with higher odds of major depression [OR: 1.15, 95% CI: 1.03, 1.29] and current depression [OR: 1.57, 95% CI: 1.11, 2.23] in all individuals. The points estimates were trending in the same direction in men and women separately, although in men the confidence intervals crossed the null (Supplementary Material, Table S3).

One-sample MR provided limited evidence for a relationship between higher genetically instrumented BMI and GAD or GAD severity (Supplementary Material, Table S3).

One-sample MR provided evidence that higher BMI caused lower well-being scores in all individuals and in men and women separately (Supplementary Material, Table S6). In all individuals, a 1-SD higher genetically instrumented BMI was associated with a 0.22 reduction in well-being score [95%CI: −0.32, −0.13].

**Figure 1:**
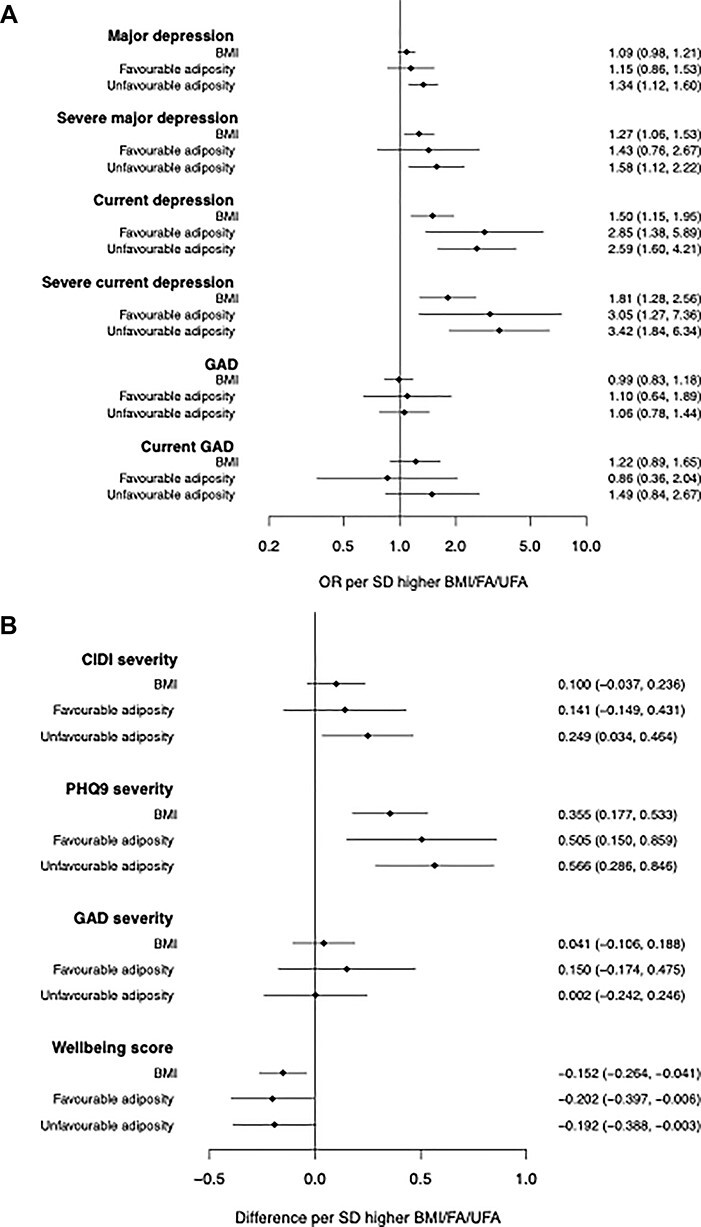
Two-sample Mendelian randomization IVW results for BMI, favourable and unfavourable adiposity in UK Biobank representing (**A**) odds of the binary mental health outcome per standard deviation change in genetically determined BMI, 95% confidence interval in brackets and (**B**) betas representing standard deviation change in the continuous mental health outcome per standard deviation change in genetically determined BMI, 95% confidence interval in brackets.

Two-sample MR provided further evidence that higher genetically instrumented BMI is associated with depression outcomes (Fig. 1 and Table 3). For example, a 1-SD higher BMI caused 1.50 [95%CI: 1.15, 1.95] and 1.09 [95%CI: 0.98, 1.21] higher odds of current depression and major depression, respectively. Results also suggest that higher BMI is associated with more severe depression (Fig. 1 and Table 3). For example, a 1-SD higher BMI caused 1.81 [95%CI: 1.28, 2.56] higher odds of severe current depression and 1.27 [95%CI: 1.06, 1.53] higher odds of severe major depression. The effect estimates tended to be higher in women, but confidence intervals overlapped.

**Table 3:** Two-sample Mendelian randomization results in UK Biobank

			BMI		Favourable adiposity		Unfavourable adiposity	
**Mental health outcome**	**Strata**	**N cases (controls)**	**OR (95% CI) per SD higher BMI**	***P***	**OR (95% CI) per SD higher FA**	***P***	**OR (95% CI) per SD higher UFA**	***P***
Major depression	All	34 739 (110844)	1.09 (0.98, 1.21)	1.12E−01	1.15 (0.86, 1.53)	3.52E−01	1.34 (1.12, 1.60)	2.95E−03
	Males only	10 808 (52615)	1.04 (0.89, 1.21)	6.36E−01	1.15 (0.73, 1.81)	5.50E−01	1.41 (1.04, 1.91)	3.25E−02
	Females only	23 931 (58229)	1.12 (0.99, 1.27)	7.89E−02	1.14 (0.80, 1.63)	4.83E−01	1.32 (1.09, 1.59)	6.93E−03
Severe major depression	All	5483 (140100)	1.27 (1.06, 1.53)	1.35E−02	1.43 (0.76, 2.67)	2.73E−01	1.58 (1.12, 2.22)	1.21E−02
	Males only	1441 (61982)	1.38 (0.96, 1.98)	8.96E−02	2.31 (0.88, 6.06)	9.63E−02	1.94 (1.02, 3.69)	5.05E−02
	Females only	4042 (78118)	1.23 (1.00, 1.52)	5.92E−02	1.23 (0.58, 2.58)	5.93E−01	1.48 (1.00, 2.21)	5.98E−02
Current depression	All	2641 (143026)	1.50 (1.15, 1.95)	3.81E−03	2.85 (1.38, 5.89)	7.68E−03	2.59 (1.60, 4.21)	4.49E−04
	Males only	962 (62500)	1.35 (0.89, 2.06)	1.67E−01	6.41 (1.94, 21.14)	4.33E−03	1.60 (0.72, 3.54)	2.56E−01
	Females only	1679 (80526)	1.56 (1.10, 2.21)	1.59E−02	1.77 (0.71, 4.40)	2.29E−01	3.33 (1.70, 6.54)	1.25E−03
Severe current depression	All	1787 (143880)	1.81 (1.28, 2.56)	1.23E−03	3.05 (1.27, 7.36)	1.78E−02	3.42 (1.84, 6.34)	3.86E−04
	Males only	662 (62800)	1.92 (1.15, 3.19)	1.49E−02	6.98 (1.65, 29.50)	1.23E−02	2.36 (0.90, 6.21)	9.09E−02
	Females only	1125 (81080)	1.75 (1.14, 2.68)	1.20E−02	1.87 (0.61, 5.68)	2.79E−01	4.29 (2.01, 9.19)	6.01E−04
GAD	All	7218 (89440)	0.99 (0.83, 1.18)	9.04E−01	1.10 (0.64, 1.89)	7.23E−01	1.06 (0.78, 1.44)	7.30E−01
	Males only	2533 (43739)	1.01 (1.00, 1.02)	1.74E−01	1.03 (0.99, 1.08)	1.74E−01	1.00 (0.98, 1.03)	8.11E−01
	Females only	4685 (45701)	0.88 (0.72, 1.08)	2.13E−01	0.89 (0.45, 1.74)	7.26E−01	1.08 (0.74, 1.57)	7.02E−01
Current GAD	All	1844 (94798)	1.22 (0.89, 1.65)	2.17E−01	0.86 (0.36, 2.04)	7.27E−01	1.49 (0.84, 2.67)	1.84E−01
	Males only	646 (45622)	1.65 (0.99, 2.76)	6.01E−02	1.39 (0.32, 5.93)	6.63E−01	1.60 (0.60, 4.21)	3.51E−01
	Females only	1198 (49176)	1.02 (0.69, 1.49)	9.32E−01	0.60 (0.20, 1.77)	3.59E−01	1.41 (0.68, 2.94)	3.64E−01
**Mental health outcome**	**Strata**	**N total**	***β* (95% CI) per SD higher BMI**	***P***	***β* (95% CI) per SD higher FA**	***P***	***β* (95% CI) per SD higher UFA**	***P***
CIDI severity	All	145 668	0.100 (0.070)	1.56E−01	0.141 (0.148)	3.48E−01	0.249 (0.110)	2.94E−02
	Males only	63 462	0.052 (0.079)	5.13E−01	−0.050 (0.230)	8.30E−01	0.101 (0.152)	5.11E−01
	Females only	82 206	0.139 (0.093)	1.40E−01	0.293 (0.205)	1.60E−01	0.366 (0.130)	7.84E−03
PHQ9 severity	All	145 668	0.355 (0.091)	2.06E−04	0.505 (0.181)	8.49E−03	0.566 (0.143)	3.23E−04
	Males only	63 462	0.245 (0.109)	2.73E−02	0.534 (0.263)	5.01E−02	0.169 (0.202)	4.09E−01
	Females only	82 206	0.432 (0.117)	4.31E−04	0.508 (0.249)	4.86E−02	0.865 (0.173)	1.43E−05
GAD severity	All	145 069	0.041 (0.075)	5.85E−01	0.150 (0.166)	3.71E−01	0.002 (0.125)	9.86E−01
	Males only	63 246	0.074 (0.083)	3.80E−01	0.309 (0.230)	1.89E−01	−0.070 (0.166)	6.74E−01
	Females only	81 823	0.009 (0.103)	9.33E−01	0.042 (0.234)	8.60E−01	0.043 (0.162)	7.94E−01
Well-being score	All	141 447	−0.152 (0.057)	9.18E−03	−0.202 (0.100)	5.07E−02	−0.192 (0.100)	6.11E−02
	Males only	61 423	−0.084 (0.072)	2.49E−01	−0.068 (0.150)	6.56E−01	0.017 (0.148)	9.08E−01
	Females only	80 024	−0.204 (0.068)	3.54E−03	−0.312 (0.133)	2.53E−02	−0.349 (0.097)	9.33E−04

Results from the inverse variance weighted instrumental variable analysis (IVW). BMI = body mass index, FA = favourable adiposity, UFA = unfavourable adiposity. *β* represents standard deviation change in mental health outcome for standard deviation change in genetically determined adiposity trait, 95% confidence interval in brackets.

Genetically higher BMI was not associated with higher odds of GAD or GAD severity (Fig. 1 and Table 3), while higher genetic BMI was associated with lower well-being scores in all individuals and women only (Fig. 1 and Table 3). In all participants, a 1-SD higher genetically instrumented BMI caused a 0.15 reduction in well-being score [95%CI: −0.26, −0.04].

The effects of higher BMI on mental health outcomes were directionally consistent when more pleiotropy robust two-sample MR methods were utilized (Supplementary Material, Table S4). MR-Egger provided no evidence of horizontal pleiotropy.

### Sensitivity analyses

Using one-sample MR approaches in the unrelated subset, we repeated our analyses excluding individuals on antidepressant medications (Supplementary Material, Table S5, Fig. S1A, S2A). Results were similar for depression and GAD outcomes when excluding individuals on antidepressants at recruitment to the UK Biobank study (Supplementary Material, Table S5). For example, a 1-SD higher genetically instrumented BMI is associated with 1.14 [95%CI 1.02, 1.29] higher odds of major depression and 1.65 [95%CI: 1.08, 2.52] higher odds of current depression.

We performed one-sample MR in the unrelated subset looking at (a) atypical depression cases (*n* = 2632) only versus controls and (b) major depression in the absence of atypical depression cases (*n* = 29 379) versus controls. These analyses demonstrated that genetically instrumented higher BMI was robustly associated with atypical depression, with a 1-SD higher BMI causing 2.21 [95%CI: 1.59, 3.09] higher odds of atypical depression. This was consistent in sex-stratified analyses (Supplementary Material, Table S3). In our major depression analyses excluding atypical cases, we observed an attenuation of the OR, with a 1-SD higher BMI associated with 1.09 higher odds of major depression [95%CI: 0.97, 1.23]. In sex-stratified analyses, the effect was attenuated to the null in men but tentatively remained in women (Supplementary Material, Table S3).

### Favourable adiposity versus unfavourable adiposity and mental health

One-sample MR provided evidence that higher genetically instrumented favourable adiposity was associated with higher current depression in all individuals [OR: 2.48, 95% CI: 1.27, 4.84] (Supplementary Material, Table S3). In sex-stratified analyses, the effect estimates were directionally consistent although in women the confidence intervals crossed the null. MR using the unfavourable adiposity variants provided evidence for a causal role of higher unfavourable adiposity on depression. A genetically determined 1-SD higher UFA was associated with 1.23 higher odds of major depression [95% CI: 1.08, 1.41] and 2.10 higher odds of current depression [95% CI: 1.37, 3.21]. The point estimates were consistent in men and women; however, the confidence intervals were much wider in men (Supplementary Material, Table S3).

Two-sample MR using both the favourable and unfavourable adiposity variants provided similar results. Higher favourable and unfavourable adiposity was associated with higher odds of depression, with stronger associations for more severe depression phenotypes (Fig. 1 and Table 3).

Exclusion of individuals taking antidepressants at baseline did not alter our findings (Supplementary Material, Table S5, Fig. S1B and C, S2B and C). Atypical depression was associated with higher favourable and unfavourable adiposity in all individuals (Supplementary Material, Table S3) and exclusion of atypical depression cases from our major depression variable did not alter our findings in the one-sample setting (Supplementary Material, Table S3).

Neither one- nor two-sample MR provided evidence for a relationship between both favourable and unfavourable adiposity and GAD or GAD severity (Supplementary Material, Table S3).

In contrast, both the favourable and unfavourable adiposity variants were associated with lower well-being scores (Table 3 and Supplementary Material, Table S3). In sex-stratified analyses, the favourable and unfavourable adiposity variants were only associated with lower well-being in women (−0.31 [95%CI; −0.57, −0.05] in women and −0.068 [95%CI: −0.36, 0.23] in men).

For all two-sample MR analyses, MR methods that are more robust to pleiotropy provided consistent results and MR-Egger provided no evidence of horizontal pleiotropy (Supplementary Material, Tables S6 and S7).

### Non-linear relationships

**Figure 2:**
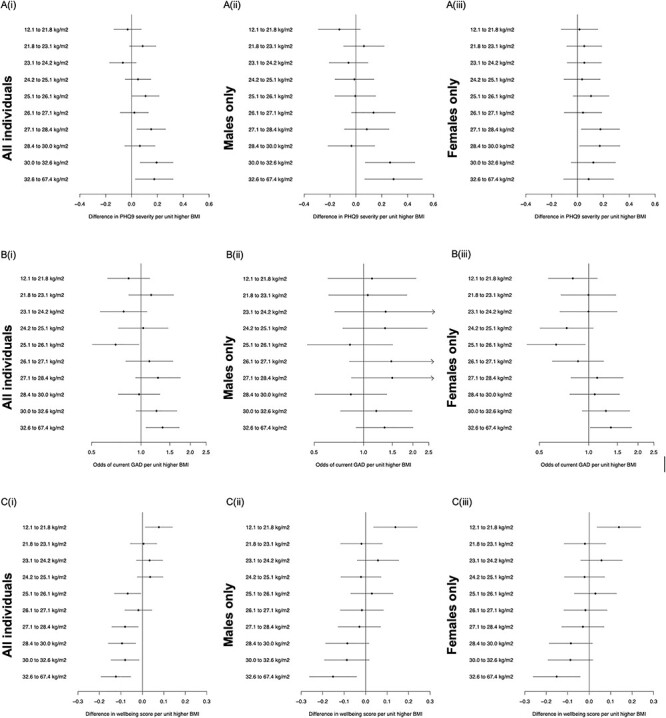
Summary of the results from the non-linear Mendelian randomization for (**A**) PHQ9 severity, (**B**) current GAD and (**C**) well-being score. Betas (continuous) and odds ratios (binary) represent the difference in mental health outcome per unit higher BMI. Results are presented for all individuals and male and females separately.

There was some evidence that both low and high BMI resulted in higher PHQ9 severity scores in males only (Fig. 2, Supplementary Material, Table S8). For men, a unit lower BMI in the lowest BMI decile (<21.8 kg/m^2^) was associated with a higher PHQ9 severity score [ß: 0.13, 95%CI: 0.03, 0.29], while in the highest BMI decile (>32.5 kg/m^2^), a unit higher BMI was associated with higher PHQ9 severity [ß: 0.29, 95%CI: 0.07, 0.51].

Non-linear MR provided tentative evidence for a non-linear relationship between higher BMI and current GAD in women but not in men (Fig. 2, Supplementary Material, Table S8). For women in the lowest BMI decile (<21.8 kg/m^2^), a unit lower BMI was associated with 1.25 [95%CI: 0.88, 1.75] higher odds of current GAD. For women in the highest BMI decile (32.6 to 67.4 kg/m^2^), a unit higher BMI was associated with 1.37 [95%CI: 1.02, 1.83] higher odds of current GAD.

The strongest evidence of non-linear relationships was found between BMI and well-being, with both low and high BMI associated with lower well-being (Fig. 2 and Supplementary Material, Table S8). For all individuals in the lowest BMI decile (<21.8 kg/m^2^), lower BMI was associated with lower well-being [ß: −0.08, 95%CI: −0.14, 0.01], while for individuals in the highest BMI decile (>32.5 kg/m^2^), a unit higher BMI was also associated with lower well-being [ß: −0.12, 95%CI: −0.19, −0.05]. The same non-linear relationship was seen when stratified by sex although the confidence intervals in females crossed the null.

## Discussion

Using Mendelian randomization and well-validated mental health measures in the UK Biobank, our study provides further evidence that higher BMI, and therefore obesity, leads to higher odds of depression (8–10,12) and higher depression severity and lowers well-being (16,17). There was evidence that both high (>32.5 kg/m^2^) and low BMI (<21.8 kg/m^2^) may lead to lower well-being in both men and women. In addition, we tested which component of higher adiposity (psychological/adverse social effect of excess weight or metabolic pathways) causes the higher risk of mental health outcomes. Using this approach, we provide evidence that higher adiposity in the absence of an adverse metabolic health profile causes depression and lowers well-being. In contrast, we found little evidence that higher adiposity in the presence or absence of adverse metabolic consequences causes generalized anxiety disorder.

The pathways from BMI to mental health could be biological or social. The biological pathways include the role of BMI as a risk factor for other negative health outcomes, such as diabetes and cardiovascular disease. In contrast societal influences, perceptions and stigma could cause individuals to associate negative health consequences with higher BMI and consequently report poorer mental health. To explore this further, we used two sets of genetic variants—one which associates with higher adiposity but better metabolic health (favourable adiposity) and the second that associates with higher adiposity but poorer metabolic health (unfavourable adiposity). The effect estimates for depression and well-being were consistent for both sets of genetic variants, suggesting that the pathway from higher BMI to adverse mental health is not purely metabolic and to some extent may be driven by other factors (e.g. psychosocial factors). This may be partially explained by the relationship between higher BMI and lower socioeconomic position (SEP) and social contact as demonstrated by ourselves (18,19) and others (20).The associations between BMI and SEP may be predominantly driven by familial effects (18,21). In combination, with the evidence in UK populations that lower SEP associates with depression (22), this may mean that our results reflect a causal pathway from higher adiposity to lower SEP to higher depression. An alternative pathway that may explain the causal relationship between higher BMI and mental health is pain. Future work should look at multivariate approaches to tease apart these associations further.

The associations between higher adiposity and higher odds of depression in this study build on previous work by ourselves (12) and others (9, 11). Here, we were able to utilize the phenotypically rich MHQ in UK Biobank, which highlights (a) a stronger relationship with current depression as defined by the PHQ-9 and (b) the importance of atypical depression in the adiposity to major depression relationship using the CIDI-SF definition. Our findings were consistent with a recent study from Kappleman using the PHQ-9 data (23) that demonstrated that genetically instrumented BMI was associated with anhedonia, tiredness, appetite changes and feelings of inadequacy in the PHQ9. Atypical depression is characterized by weight gain and sleeping more than usual. This was robustly associated with BMI, favourable and unfavourable adiposity in our analyses. As atypical depression could result in unhealthy diets and lower physical activity levels reverse causal inference needs to be assessed.

This study provides further evidence of the adverse effects of higher BMI on well-being, which builds on previous work in the UK Biobank focused on subjective well-being (16). The previous work by Wooton *et al.* highlighted that the relationship between higher BMI and subjective well-being was predominantly driven by the health satisfaction component included in their subjective well-being measure. Here, we have used the MHQ measure of subjective well-being that incorporates general happiness, general happiness with own health and belief that one’s own life is meaningful. Our findings are similar to those of the previous study with higher BMI lowering well-being, although our non-linear analyses provide evidence that both high and low BMI can have adverse effects on well-being.

Happiness is generally highly valued by individuals (24) and has the potential to act as a motivator in tackling the rising prevalence of obesity. Further work needs to explore whether emphasizing the potential benefits to mental health and well-being that could be achieved by weight loss is a better motivator for weight loss than the well-established adverse physical health consequences of obesity.

This study provided limited evidence for the causal role of adiposity in GAD. Previous observational studies have provided mixed evidence for the role of higher BMI in GAD, with some demonstrating positive associations (25,26), while others provided no evidence of an association (2) or highlight age, sex and racial differences (27). Observationally, we observed strong associations with GAD, but not when we adjusted our observational models for depression or when we used MR, which provided limited evidence that higher adiposity causes GAD. Non-linear MR suggested high and low BMI in women may cause GAD and this fits with previous observational analyses where heterogeneous associations with BMI were observed to be potentially influenced by demographic characteristics (27).

The adverse effects of low BMI on well-being suggested by our non-linear MR analyses were of similar magnitude to the adverse effects of higher BMI. However, the effects of higher BMI were seen across a wider range of BMIs, in larger numbers of people, and so, if real will have greater societal implications. The majority of individuals in the low BMI group were actually within the ‘recommended’ range (18.5–24.9 kg/m^2^). Only 2000 individuals in the UK Biobank were with a BMI in the underweight category (BMI < 18.5 kg/m^2^), meaning we had insufficient power to apply any MR to this subgroup. There was limited evidence for non-linear findings in other outcomes, although there was some evidence that males in the lowest BMI decile with lower BMI had higher PHQ9 scores and females in the lowest BMI decile with lower BMI were more likely to report GAD. Previous research has suggested a U-shaped relationship between BMI and GAD in women (27), which could be driven by eating disorders (e.g. anorexia nervosa).

The individual level data available in UK Biobank allowed us to stratify our analyses by sex and exclude individuals on antidepressant medication. In general our findings were similar when analyses were stratified by sex and when we excluded individuals taking antidepressant medication. The non-linear MR provided some suggested sex differences, with evidence of non-linear relationships between (a) the PHQ-9 and higher BMI in men, but not in women and (b) current GAD in women only. However, larger sample sizes are required to confirm these sex-specific findings.

### Strengths and limitations

The major strength of this study was the availability of individual-level data in 145 668 individuals with well-validated mental health outcomes available. This allowed us to perform several stratified analyses (e.g. sex and antidepressant usage stratified) and to run several sensitivity analyses including non-linear MR to test for non-linear causal relationships between adiposity and mental health outcomes. We acknowledge several limitations with this study. First, the UK Biobank is not population representative. However, our results were consistent with several other studies that use data from different age ranges and from different European countries. Second, these analyses focused on a European population, so our findings are not generalizable to other populations. Third, the mental health questionnaire was only available in a subset of Biobank participants and work by ourselves and others have suggested potential participation biases in this subset (28). Fourth, the favourable and unfavourable instruments only explain a small percentage of variation in body fat percentage, limiting our power; however, the large numbers of individuals with mental health questionnaire data mean that we had sufficient power to detect the OR reported in the observational analyses. Fifth, the sensitivity analyses were performed in the one-sample MR framework in the unrelated subset, which may not fully account for population structure. However, these findings were consistent with the two-sample MR approaches. Finally, while the favourable adiposity variants associate with a more favourable metabolic profile, they do associate with higher C-reactive protein (CRP), which means in these analyses we cannot rule out the role of inflammation in linking adiposity to mental health outcomes.

## Conclusion

In summary, using well-validated mental health measures in up to 145 668 UK Biobank participants, we provide evidence that higher adiposity in the presence and absence of adverse metabolic effects, as estimated by genetics, is causal to higher odds of depression and lower well-being scores. Our findings add to the evidence base to support the need to reduce obesity because of the adverse consequences on depression and well-being.

## Materials and Methods

### UK Biobank

The UK Biobank recruited over 500 000 adults aged between 37 and 73 years of age from 2006 to 2010. The study is extensively described elsewhere (29). Briefly, extensive phenotypic data (from questionnaires, anthropometric measures, etc.) were collected at baseline and subjects agreed to have their health followed over time and participate in subsequent follow-up activities. All participants were asked to provide blood, urine and saliva samples, which were used for subsequent analyses. Genetic data were available for all participants and SNP genotypes were generated from the Affymetrix Axiom UK Biobank array (~450 000 individuals) and the UKBiLEVE array (~50 000 individuals). The genetic data underwent extensive centralized quality control (30). This study includes 145 668 individuals with mental health questionnaire data and measured BMI available, who were defined European using principal component analysis as previously described (12).

### Exposure and outcome measures

#### Body mass index (BMI) and body fat percentage (BFP)

BMI was calculated for all participants from measured weight (kg)/height (m)^2^. Body fat was calculated by impedance measurement (variable 23 099). Both BMI and body fat percentage were inverse normalized prior to analysis.

#### Mental health outcomes

Mental health outcomes were defined using the definitions summarized in Davis *et al*. (31) and the R code that is freely available (https://data.mendeley.com/datasets/kv677c2th4/3). Here we focused on:

1. Major depression and major depression severity (CIDI severity)
2. Current depression and current depression severity (PHQ9 severity)
3. Generalized anxiety disorder (GAD) and anxiety severity
4. Current anxiety (Current GAD)
5. Well-being

More details of the coding and variables used are provided in the supplementary material.

### Observational associations

Mental health outcomes were regressed against BMI and body fat percentage using logistic (major depression, severe major depression, current depression, severe current depression, GAD and current GAD) and linear regression (CIDI severity, PHQ9 severity, GAD severity and well-being score) models. All models were adjusted for age at baseline, sex and assessment centre, the Townsend Deprivation Index (TDI; variable 189) and smoking status then further adjusted for type 2 diabetes, alcohol intake, physical activity, hypertension, low-density lipoprotein (LDL), high-density lipoprotein (HDL), cardiovascular disease (CVD) and coronary artery disease (CAD). As depression and GAD are both highly heterogeneous conditions and are often highly correlated, we adjusted our analyses for depression with GAD and vice versa.

### Genetic variants

Well imputed (INFO score ≥ 0.9) genetic variants were selected from the UK Biobank’s imputation data for BMI, favourable adiposity and unfavourable adiposity (Supplementary Material, Table S9).

### BMI

Genetic variants associated with BMI at genome-wide significance (*P* < 5 × 10^−8^) in the GIANT consortium of up to 339 224 people of European ancestry were selected (32). UK Biobank samples did not contribute to this meta-analysis. The full list of variants included are summarized in Supplementary Material, Table S9. These variants explained 1.6% of the variance in BMI in the UK Biobank (Supplementary Material, Table S10).

### Favourable and unfavourable adiposity variants

We selected 36 favourable adiposity variants and 38 unfavourable adiposity variants (manuscript currently under review). These variants were associated (at *P* < 5 × 10^−8^) with body fat percentage and a composite metabolic phenotype consisting of body fat percentage, HDL-cholesterol, triglycerides, sex hormone–binding globulin (SHBG), alanine transaminase and aspartate transaminase. While both sets of variants are associated with higher adiposity, the unfavourable variants are associated with lower HDL-cholesterol, lower SHBG and higher triglycerides and liver enzymes; the favourable adiposity variants are paradoxically associated with higher HDL-cholesterol, higher SHBG and lower triglycerides and liver enzymes. The favourable and unfavourable adiposity variants explained 0.2% and 0.6% variance in body fat percentage and 0.1% and 0.9% variance in BMI, respectively, in the UK Biobank (Supplementary Material, Table S10).

### One-sample Mendelian randomization

We employed the two-stage least-squares regression estimator method that uses predicted levels of BMI/FA/UFA per genotype and regresses the mental health outcome against these predicted values. First, we calculated the association between the BMI, FA or UFA GRS and BMI or BFP, respectively. These predicted values were then used as the independent variable and the mental health and well-being measures as the dependent variables in a logistic (binary) or linear (continuous) regression model.

### Two-sample Mendelian randomization

Firstly, we performed GWAS of the 10 mental health outcomes, using BOLT-LMM (33) and adjusting for age, sex and genotyping platform.

Two-sample MR was performed in R (version 3.5.0), by extracting the genetic variants for (a) BMI, (b) favourable adiposity and (c) unfavourable adiposity from BOLT-LMM (33) GWAS analyses for the 10 mental health outcomes. We next harmonized the direction of effects between the adiposity raising exposure and our mental health outcomes, where for each variant, the exposure allele was associated with higher adiposity.

For each SNP individual effect-estimates were calculated using the Wald ratio, by dividing the SNP-outcome association by the SNP-exposure association. Random-effects inverse variance weighted (IVW) meta-analysis method was then used to combine the individual variants into a single instrument.

For binary outcomes, we computed odds ratios (OR), which represent the change in odds of our outcome per SD higher genetically instrumented BMI (SD ~ 4.6 kg/m^2^) or body fat percentage (SD ~ 8.4%).

In the absence of horizontal pleiotropy or when horizontal pleiotropy is balanced, the IVW method provides an unbiased effect estimate (34). Several sensitivity analyses were performed to evaluate the potential for unbalanced (directional) horizontal pleiotropy. We calculated the proportion of variance explained and the F-statistic (an F-statistic of < 10 is indicative of weak instrument bias). Three further MR methods were used and compared to account for directional pleiotropy: MR Egger (35), weighted median and penalized weighted median (36). The weighted median stipulates that at least 50% of the weight in the analysis stems from variants that are valid instruments (36). The penalized weighted median is equivalent to the weighted median method but downweights the contribution to the analysis of heterogeneous genetic variants identified by Cochran’s Q statistic (36).MR-Egger can provide unbiased estimates even when all SNPs violate the exclusion restriction assumption (i.e. they affect the outcome by means other than via the risk factor of interest). However, to use MR-Egger, there must be negligible measurement error (NOME) in the genetic instrument and the Instrument Strength Independent of Direct Effect (InSIDE) assumption must be satisfied (35).

### Non-linear Mendelian randomization

To explore non-linear relationships, we employed non-linear MR, using the *nlmr* package in R (https://github.com/jrs95/nlmr) (37). This package regresses the exposure (here, BMI) on the instrumental variable (genetic risk score for BMI) to generate the ‘IV-free’ exposure (non-genetic component of BMI). In strata of the IV-free exposure, the local average causal effect (LACE) of BMI on the outcome is estimated as a ratio of coefficients: the IV association with the outcome divided by the IV association with the exposure. This approach assumes a linear effect of the IV on the exposure. The *nlmr* package provides two options for estimating the non-linear effects of an exposure on an outcome; fractional polynomials and a piecewise linear function. Fractional polynomial methods can be unduly influenced by the extremes of a distribution; therefore, we used the piecewise linear function only. The piecewise linear method estimates a continuous function, whereby a linear relationship is fitted within each stratum of the IV-free exposure distribution, constrained so that each segment begins where the previous one ended. Confidence intervals are estimated by bootstrapping. We a priori selected to run our analysis across deciles of IV-free BMI. Two statistical tests of non-linearity are presented: Cochran’s Q statistic assesses whether heterogeneity of LACE estimates is greater than would be expected by chance, and a quadratic test metaregresses the LACE estimates against the mean exposure value in each stratum (equivalent to fitting a quadratic exposure-outcome model). These analyses were performed in the unrelated subset only, as the *nlmr* package in R cannot account for relatedness.

### Sensitivity analyses

We repeated our analyses in an unrelated subset using one-sample MR approaches to firstly confirm findings between BMI and depression and GAD outcomes in individuals not taking antidepressant medication. We excluded individuals on antidepressants to test whether the BMI-depression association was driven by their usage. The unrelated subset was defined using the KING Kinship matrix to separate out related individuals (up to third degree) and included 123 923 individuals with MHQ data available. Antidepressant medication was coded using 82 relevant medication codes in UK Biobank (Supplementary Material, Table S11 and field 20 003, http://biobank.ndph.ox.ac.uk/showcase/coding.cgi?id=4&nl=1) from the study interview undertaken by a trained nurse (31). This variable represents treatment at baseline interview and not lifetime treatment.

We also tested the causal relationship between adiposity and depression in atypical depression cases, to test whether any adiposity association with depression is solely driven by atypical depression cases, which by definition involves weight gain. Atypical depression was coded from our major depression variable and was defined as depression with weight gain (field 20 526) and sleeping too much (field 20 534) (38).

## Supplementary Material

Online_supplement_HMG_R1_ddab204
