# Supplementary material for: Higher adiposity and mental health: causal inference using Mendelian randomization
Source: Hum Mol Genet. 2021 Jul 16;30(24):2371–82. doi: 10.1093/hmg/ddab204 (PMC8643500; doi:10.1093/hmg/ddab204)
Supplement: Online_supplement_HMG_R1_ddab204 [file online_supplement_hmg_r1_ddab204.docx]

**Index supplementary material:**

Supplimetary methods Page 2

Supplementary table 1 Page 5

Supplementary table 2 Page 16

Supplementary table 3 Page 8

Supplementary table 4 Page 9

Supplementary table 5 Page 10

Supplementary table 6 Page 11

Supplementary table 7 Page 12

Supplementary table 8 Page 13

Supplementary table 9 Page 14

Supplementary table 10 Page 18

Supplementary table 11 Page 19

Supplementary figure 1A Page 21

Supplementary figure 1B Page 22

Supplementary figure 1C Page 23

Supplementary figure 2A Page 24

Supplementary figure 2B Page 25

Supplementary figure 2C Page 26

**Supplementary Methods: Outcome phenotype creation**

In the subset of unrelated individuals (N = 145,668) with the MHQ data available we defined 4 continuous variables and 6 binary variables for depression, anxiety and wellbeing using the well-established and validated Composite International Diagnostic Interview Short Form (CIDI-SF) and the Patient Health Questionnaire-9 (PHQ9), replicating the methods previously described by Davis et al. The 4 continuous variables were: severity of lifetime depression (**CIDI severity**), severity of current depression (**PHQ9 severity**), severity of anxiety (**GAD severity**) and wellbeing (**Wellbeing score**). The 6 binary variables were: **Major depression, severe major depression, current depression, severe current depression, generalized anxiety (GAD), current GAD**.

*Continuous variables*

All binary continuous variables were derived according to the methods previously described by Davis et al. More detailed description of phenotype derivation can be found in supplementary material from Davis et al. and the detailed R code used can be freely downloaded here: <https://data.mendeley.com/datasets/kv677c2th4/3>.

We created a severity of lifetime depression variable using 8 variables from the CIDI-SF (**CIDI severity**). In each case (unless specifically stated) the options were Yes (score of 1) or No (score of 0), replicating the methods previously described by Davis et al:

- Have you ever had a time in your life when you felt sad, blue, or depressed for two weeks or more in a row? (data field 20446)
- Have you ever had a time in your life lasting two weeks or more when you lost interest in most things like hobbies, work, or activities that usually give you pleasure? (data field 20441)
- Did you feel more tired out or low on energy than is usual for you? (data field 20449)
- Did you gain or lose weight without trying, or did you stay about the same weight? (data field 20536). Here any response other than stayed the same, resulted in adding one to the overall CIDI-SF response variable.
- Did your sleep change? (data field 20532)
- Was that: [re sleep change] Waking too early? (data field 20535)
- Did you have a lot more trouble concentrating than usual? (data field 20435)
- Did you think a lot about death - either your own, someone else's or death in general? (data field 20437)

We created a severity of current depression variable by adding 9 items from the PHQ9 (**PHQ9 severity**), replicating the methods previously described by Davis et al. The items were the answers to “Over the last 2 weeks, how often have you been bothered by any of the following problems (depression symptoms)?”:

- Little interest or pleasure in doing things (data field 20514).
- Feeling bad about yourself or that you are a failure or have let yourself or your family down (data field 20507).
- Feeling down, depressed, or hopeless (data field 20510).
- Trouble concentrating on things, such as reading the newspaper or watching television (data field 20508).
- Trouble falling or staying asleep or sleeping too much (data field 20517).
- Moving or speaking so slowly that other people could have noticed? Or the opposite - being so fidgety or restless that you have been moving around a lot more than usual (data field 20518).
- Feeling tired or having little energy (data field 20519).
- Thoughts that you would be better off dead or of hurting yourself in some way (data field 20513).
- Poor appetite or overeating (data field 20511).

For each item we scored 1 if the answer was “several days”, 2 if the answers was “more than half the days” and 3 if the answer was “nearly every day”.

We created a severity of anxiety variable by adding 7 items from the mental health questionnaire (**GAD severity**), replicating the methods previously described by Davis et al. The items were the answers to “Over the last 2 weeks, how often have you been bothered by any of the following problems (anxiety symptoms)?

- Feeling nervous, anxious or on edge (item 20506)
- Not being able to stop or control worrying (item 20509)
- Worrying too much about different things (item 20520)
- Trouble relaxing (item 20515)
- Being so restless that it is hard to sit still (item 20516)
- Becoming easily annoyed or irritable (item 20505)
- Feeling afraid as if something awful might happen (item 20512)

For each item we scored 1 if the answer was “several days”, 2 if the answers was “more than half the days” and 3 if the answer was “nearly every day”.

We created a wellbeing variable by adding 3 items from the mental health questionnaire (**Wellbeing score**), replicating the methods previously described by Davis et al. Two questions provide a euthymic (‘positive emotion’) aspect of wellbeing and one from the WHO-Quality Of Life (WHOQOL) provides a ‘meaning’ (eudemonic) measure of wellbeing. The 3 items were:

- In general how happy are you? (data field 20458).
- In general how happy are you with your health? (data field 20459).

For each of these 2 items we scored 1 if the answer was “extremely unhappy”, 2 if “very unhappy”, 3 if “moderately unhappy”, 4 if “moderately happy”, 5 if “very happy” and 6 if “extremely happy”.

- To what extent do you feel your life to be meaningful? (data field 20460).

For this item we scored 1 if the answer was “not at all”, 2 if “a little”, 3 if “a moderate amount”, 4 if “very much” and 5 if “an extreme amount”.

*Binary variables*

All binary variables were derived according to the methods previously described by Davis et al. More detailed description of phenotype derivation can be found in supplementary material from Davis et al. and the detailed R code used can be freely downloaded here: https://data.mendeley.com/datasets/kv677c2th4/3.

The severity of lifetime depression variable was then utilised to create a binary variable (**Major depression**), where cases were defined based on having at least one core symptom of depression, most or all of the day on most or all days for a two week period, with at least five depressive symptoms that represent a change from usual occurring over the same time-scale, with some or a lot of impairment. Fields and codes used to derive this variable where:

- Persistent sadness (20446) = Yes OR Loss of interest (20441) = Yes

AND

- How much of day (20436) = Most of day or All day long

AND

- Did you feel this way (20439) = Almost every day or Every day

AND

- Impairment (20440) = Somewhat or A lot

AND

- Total number of symptoms endorsed >= 5
  - Persistent sadness (core) 20446; Loss of interest (core) 20441; Tired or low energy 20449; Gain or loss of weight 20536 = Gain, Loss or Gain and loss; Sleep change 20532; Trouble concentrating 20435; Feeling worthless 20450; Thinking about death 20437

A subgroup of severely affected participants (**Severe major depression**) was defined from Major depression when:

- Scored 8 in the severity of lifetime depression variable.
- Responded “a lot” to data field 20440 (Impact on normal roles during worst period of depression).

Major depression and Severe major depression controls were defined from the subset who had undertaken the MHQ not endorsing depression or screening positive on PHQ or CIDI. Fields and codes used to derive this variable where:

- NOT (reported diagnosis of depression 20544 or 20002)

AND

- NOT symptoms from above case derivation

AND

- PHQ score ≤5

This severity of current depression and Major depression variables were then utilised to create a binary current depression variable (**Current depression**), where cases were defined based on the following criteria:

- Individuals who were cases for Major depression and answered “several days” or “more than half the days” or “nearly every day“ in data fields 20514 and 20510 and scored “more than half the days” or “nearly every day“ in at least 4 of the severity of current depression variable items.

A subgroup of severely affected participants (**Severe current depression**) was defined from Current depression when:

- Scored 15 or more in the severity of current depression variable.

Current depression and Severe current depression controls were defined from the subset who had undertaken the MHQ and who:

- Scored less than 5 in the severity of current depression variable.

AND

- No self-reported depression and anxiety

AND

- No record of depression in hospital episode data

AND

- No known use of antidepressants

The severity of anxiety variable was used to derive lifetime generalized anxiety disorder (**GAD**) by combining it with further questions from the MHQ. Participants were defined as cases if:

- Responded yes to the question: “Have you ever had a period lasting one month or longer when most of the time you felt worried, tense, or anxious?” (item 20421)

AND

- Answered “all my life/as long as I can remember” OR 6 or more years to the follow up question ”What is the longest period of time that this kind of worrying has ever continued?” (item 20420)

AND

- Responded yes to the question “"People differ a lot in how much they worry about things. Did you ever have a time when you worried a lot more than most people would in your situation?" (item 20425) AND in response to the follow up question "Please think of the period in your life when you have felt worried, tense, anxious, or more worried than most people would in your situation. This could be in the past, or it could be continuing now” answered:
  - Yes to “Did you worry most days?" (item 20538) OR
  - Yes to “During that period, was your worry stronger than in other people?" (item 20542) AND answered “more than one thing” to “Did you usually worry about one particular thing, such as your job security or the failing health of a loved one, or more than one thing?" (item 20543) OR
  - Yes to “Did you ever have different worries on your mind at the same time?" (item 20540) AND answered yes to “Did you find it difficult to stop worrying?" (item 20541) OR

Controls GAD were defined as participants who:

- Responded no to the question: “Have you ever had a period lasting one month or longer when most of the time you felt worried, tense, or anxious?” (item 20421).

AND

- Had a score of less than 5 in the severity of anxiety variable.

The severity of anxiety variable was used to derive current generalized anxiety disorder (**Current GAD**) Participants were defined as cases if:

- Scored 10 or more in the 7 item GAD severity score AND were cases for the GAD binary variable.

**Supplementary table 1: Observational associations of BMI to depression adjusting for anxiety status and BMI to anxiety adjusting for depression status.**

| **Mental health outcome** | **Strata** | **Adjusted for^I^** | **OR (95% CI) per SD higher BMI** | **P** |
| --- | --- | --- | --- | --- |
| GAD | All | Usual adjustments | 1.10 (1.07, 1.13) | 4.60E-14 |
| GAD | All | Further adjusted for major depression | 1.00 (0.97, 1.02) | 0.81 |
| Current GAD | All | Usual adjustments | 1.17 (1.11, 1.22) | 6.70E-11 |
| Current GAD | All | Further adjusted for current depression | 0.98 (0.93, 1.03) | 0.41 |
| Major depression | All | Usual adjustments | 1.16 (1.14, 1.17) | <1.00E-15 |
| Major depression | All | Further adjusted for GAD | 1.16 (1.14, 1.19) | <1.00E-15 |
| Current depression | All | Usual adjustments | 1.56 (1.51, 1.62) | <1.00E-15 |
| Current depression | All | Further adjusted for current GAD | 1.51 (1.40, 1.62) | <1.00E-15 |

^I^Usual adjustments include age, sex, Townsend Deprivation Index(TDI), smoking status and assessment centre.

**Supplementary Table 2: Observational associations between higher adiposity (using BMI and body fat percentage) and 10 mental health outcomes**

|  | **BMI** | | |  |  | **Body fat percentage** | | |
| --- | --- | --- | --- | --- | --- | --- | --- | --- |
| **Mental health outcome** | **Strata** | **N cases (controls)** | **OR (95% CI) per SD higher BMI** | **P^I^** |  | **N cases (controls)** | **OR (95% CI) per SD higher body fat %** | **P^I^** |
| Major depression | All | 27,787 (90,317) | 1.14 (1.12, 1.16) | <1.00E-15 |  | 27,470 (89,284) | 1.19 (1.16 1.21) | <1.00E-15 |
|  | Males only | 8,863 (43,582) | 1.13 (1.09, 1.16) | 1.20E-14 |  | 8,737 (42,991) | 1.19 (1.15, 1.23) | <1.00E-15 |
|  | Females only | 18,924 (46,735) | 1.14 (1.12, 1.16) | <1.00E-15 |  | 18,733 (46,293) | 1.19 (1.16, 1.22) | <1.00E-15 |
| Severe major depression | All | 4,368 (113,736) | 1.30 (1.26, 1.34) | <1.00E-15 |  | 4,311 (112,443) | 1.39 (1.33, 1.46) | <1.00E-15 |
|  | Males only | 1,152 (51,293) | 1.52 (1.41, 1.64) | <1.00E-15 |  | 1,131 (50,597) | 1.59 (1.45, 1.76) | <1.00E-15 |
|  | Females only | 3,216 (62,443) | 1.25 (1.21, 1.30) | <1.00E-15 |  | 3,180 (61,846) | 1.35 (1.28, 1.42) | <1.00E-15 |
| Current depression | All | 1,989 (116,183) | 1.36 (1.30, 1.43) | <1.00E-15 |  | 1,964 (114,858) | 1.43 (1.34, 1.53) | <1.00E-15 |
|  | Males only | 728 (51,746) | 1.46 (1.33, 1.61) | 3.40E-15 |  | 716 (51,041) | 1.47 (1.30, 1.65) | 4.20E-10 |
|  | Females only | 1,261 (64,437) | 1.32 (1.25, 1.40) | <1.00E-15 |  | 1,248 (63,817) | 1.42 (1.31, 1.53) | <1.00E-15 |
| Severe current depression | All | 1,329 (116,843) | 1.43 (1.34, 1.51) | <1.00E-15 |  | 1,312 (115,510) | 1.53 (1.41, 1.65) | <1.00E-15 |
|  | Males only | 496 (51,978) | 1.54 (1.38, 1.73) | 9.00E-14 |  | 489 (51,268) | 1.57 (1.36, 1.82) | 9.10E-10 |
|  | Females only | 833 (64,865) | 1.38 (1.29, 1.48) | <1.00E-15 |  | 823 (64,242) | 1.50 (1.37, 1.66) | <1.00E-15 |
| Atypical depression | All | 2,301 (115,803) | 2.07 (1.98, 2.17) | <1.00E-15 |  | 2,277 (114,477) | 2.34 (2.20, 2.49) | <1.00E-15 |
|  | Males only | 607 (51,838) | 2.34 (2.10, 2.60) | <1.00E-15 |  | 602 (51,126) | 2.45 (2.14, 2.82) | <1.00E-15 |
|  | Females only | 1,694 (63,965) | 2.03 (1.92, 2.14) | <1.00E-15 |  | 1,675 (63,351) | 2.32 (2.17, 2.49) | <1.00E-15 |
| Major depression without atypical depression | All | 26,096 (89,707) | 1.10 (1.08, 1.12) | <1.00E-15 |  | 25,797 (88,680) | 1.14 (1.12, 1.17) | <1.00E-15 |
|  | Males only | 8,446 (43,392) | 1.09 (1.06, 1.13) | 1.60E-08 |  | 8,324 (42,802) | 1.15 (1.11, 1.20) | 2.20E-13 |
|  | Females only | 17,650 (46,315) | 1.10 (1.08, 1.12) | <1.00E-15 |  | 17,473 (45,878) | 1.14 (1.11, 1.17) | <1.00E-15 |
| GAD | All | 5,820 (73,124) | 1.06 (1.03, 1.10) | 9.60E-05 |  | 5,751 (72,306) | 1.12 (1.08, 1.17) | 2.80E-08 |
|  | Males only | 2,047 (36,346) | 1.12 (1.05, 1.18) | 3.20E-04 |  | 2,019 (35,868) | 1.18 (1.09, 1.27) | 1.30E-05 |
|  | Females only | 3,773 (36,778) | 1.05 (1.01, 1.08) | 0.018 |  | 3,732 (36,438) | 1.10 (1.05, 1.16) | 1.40E-04 |
| Current GAD | All | 1,429 (77,501) | 1.08 (1.02, 1.14) | 0.013 |  | 1,411 (76,632) | 1.11 (1.03, 1.20) | 7.00E-03 |
|  | Males only | 491 (37,898) | 1.24 (1.10, 1.39) | 3.50E-04 |  | 481 (37,402) | 1.19 (1.03, 1.38) | 0.018 |
|  | Females only | 938 (39,603) | 1.03 (0.96, 1.10) | 0.47 |  | 930 (39,230) | 1.08 (0.99, 1.19) | 0.090 |
| **Mental health outcome** | **Strata** | **N total** | **β (95% CI) per SD higher BMI** | **P^I^** |  | **N total** | **β (95% CI) per SD higher body fat %** | **P^I^** |
| CIDI severity^II^ | All | 117,676 | 0.20 (0.18, 0.22) | <1.00E-15 |  | 116,334 | 0.24 (0.21, 0.26) | <1.00E-15 |
|  | Males only | 52,259 | 0.16 (0.13, 0.19) | <1.00E-15 |  | 51,546 | 0.19 (0.15, 0.22) | <1.00E-15 |
|  | Females only | 65,417 | 0.22 (0.19, 0.24) | <1.00E-15 |  | 64,788 | 0.28 (0.25, 0.31) | <1.00E-15 |
| PHQ9 severity^II^ | All | 117,676 | 0.39 (0.36, 0.41) | <1.00E-15 |  | 116,334 | 0.46, 0.43, 0.49) | <1.00E-15 |
|  | Males only | 52,259 | 0.36 (0.32, 0.40) | <1.00E-15 |  | 51,546 | 0.38 (0.34, 0.43) | <1.00E-15 |
|  | Females only | 65,417 | 0.40 (0.37, 0.43) | <1.00E-15 |  | 64,788 | 0.52 (0.47, 0.56) | <1.00E-15 |
| GAD severity^II^ | All | 117,232 | 0.07 (0.05, 0.09) | 1.90E-10 |  | 115,897 | 0.11 (0.08, 0.14) | 2.20E-13 |
|  | Males only | 52,097 | 0.12 (0.08, 0.15) | 1.90E-11 |  | 51,388 | 0.14 (0.10, 0.18) | 8.80E-12 |
|  | Females only | 65,135 | 0.05 (0.02, 0.08) | 1.30E-03 |  | 64,509 | 0.09 (0.05, 0.12) | 1.90E-05 |
| Wellbeing Score^II^ | All | 114,542 | -0.12 (-0.13, -0.11) | <1.00E-15 |  | 113,231 | -0.15 (-0.16, -0.13) | <1.00E-15 |
|  | Males only | 50,710 | -0.08 (-0.10, -0.06) | 8.40E-13 |  | 50,014 | -0.10 (-0.13, -0.07) | 5.80E-13 |
|  | Females only | 63,832 | -0.14 (-0.15, -0.12) | <1.00E-15 |  | 63,217 | -0.18 (-0.20, -0.16) | <1.00E-15 |

^I^adjusted for age, sex, centre, TDI and smoking status and further adjusted for type 2 diabetes, alcohol intake, physical activity, hypertension, LDL, HDL, CVD and CAD

^II^Severity scores used linear regression

**Supplementary Table 3: 1-sample Mendelian randomization results in UK Biobank.**

|  |  |  | **BMI** |  |  | **FAVOURABLE ADIPOSITY** |  |  | **UNFAVOURABLE ADIPOSITY** |  |
| --- | --- | --- | --- | --- | --- | --- | --- | --- | --- | --- |
| **Mental health outcome** | **Strata** | **N cases (controls)** | **OR (95% CI) per SD higher BMI** | **P** |  | **OR (95% CI) per SD higher FA** | **P** |  | **OR (95% CI) per SD higher UFA** | **P** |
| Major depression | All | 29,594 (94,609) | 1.15 (1.03, 1.29) | 0.01 |  | 1.00 (0.81, 1.23) | 1.00 |  | 1.23 (1.08, 1.41) | 2.60E-03 |
|  | Males only | 9,222 (45,200) | 1.07 (0.88, 1.29) | 0.51 |  | 1.08 (0.77, 1.53) | 0.65 |  | 1.23 (0.95, 1.61) | 0.12 |
|  | Females only | 20,372 (49,409) | 1.20 (1.05, 1.37) | 9.10E-03 |  | 0.96 (0.73, 1.25) | 0.74 |  | 1.23 (1.05, 1.42) | 8.00E-03 |
| Severe major depression | All | 4,695 (119,508) | 1.30 (1.02, 1.66) | 0.04 |  | 1.17 (0.73, 1.86) | 0.51 |  | 1.37 (1.02, 1.85) | 0.04 |
|  | Males only | 1,231 (53,191) | 1.33 (0.82, 2.15) | 0.25 |  | 2.20 (0.93, 5.23) | 0.07 |  | 1.55 (0.80, 3.04) | 0.20 |
|  | Females only | 3,464 (66,317) | 1.29 (0.97, 1.71) | 0.08 |  | 0.91 (0.52, 1.59) | 0.74 |  | 1.32 (0.97, 1.81) | 0.08 |
| Current depression | All | 2,238 (122,036) | 1.57 (1.11, 2.23) | 0.01 |  | 2.48 (1.27, 4.84) | 7.70E-03 |  | 2.10 (1.37, 3.21) | 6.40E-04 |
|  | Males only | 816 (53,639) | 1.44 (0.80, 2.60) | 0.22 |  | 5.03 (1.75, 14.48) | 2.80E-03 |  | 1.63 (0.72, 3.68) | 0.24 |
|  | Females only | 1,422 (68,397) | 1.65 (1.07, 2.55) | 0.02 |  | 1.59 (0.67, 3.80) | 0.29 |  | 2.34 (1.44, 3.79) | 5.60E-04 |
| Severe current depression | All | 1,514 (122,760) | 1.86 (1.22, 2.83) | 4.20E-03 |  | 2.57 (1.14, 5.76) | 0.02 |  | 2.73 (1.63, 4.57) | 1.30E-04 |
|  | Males only | 565 (53,890) | 1.86 (0.92, 3.77) | 0.08 |  | 5.04 (1.42, 17.84) | 0.01 |  | 2.24 (0.84, 5.93) | 0.11 |
|  | Females only | 949 (68,870) | 1.85 (1.09, 3.14) | 0.02 |  | 1.65 (0.57, 4.75) | 0.36 |  | 2.98 (1.65, 5.36) | 2.80E-04 |
| Atypical depression | All | 2,508 (121,695) | 2.21 (1.59, 3.09) | 2.90E-06 |  | 2.23 (1.18, 4.21) | 0.01 |  | 2.28 (1.52, 3.42) | 6.80E-05 |
|  | Males only | 651 (53,771) | 2.76 (1.43, 5.33) | 2.50E-03 |  | 0.92 (0.28, 3.00) | 0.89 |  | 4.25 (1.71, 10.59) | 1.90E-03 |
|  | Females only | 1,857 (67,924) | 2.05 (1.40, 3.01) | 2.40E-04 |  | 3.14 (1.46, 6.77) | 3.50E-03 |  | 1.90 (1.24, 2.91) | 3.20E-03 |
| Major depression without atypical depression | All | 27,752 (93,942) | 1.09 (0.97, 1.23) | 0.13 |  | 0.95 (0.76, 1.18) | 0.64 |  | 1.17 (1.02, 1.35) | 0.03 |
|  | Male | 8,773 (44,998) | 1.01 (0.83, 1.24) | 0.88 |  | 1.06 (0.74, 1.51) | 0.75 |  | 1.15 (0.87, 1.51) | 0.32 |
|  | Female | 18,979 (48,945) | 1.13 (0.98, 1.31) | 0.082 |  | 0.89 (0.67, 1.18) | 0.42 |  | 1.18 (1.01, 1.38) | 0.04 |
| GAD | All | 6,163 (76,284) | 1.08 (0.86, 1.35) | 0.51 |  | 1.07 (0.70, 1.62) | 0.76 |  | 1.02 (0.78, 1.33) | 0.89 |
|  | Males only | 2,162 (37,524) | 1.44 (0.97, 2.15) | 0.07 |  | 0.92 (0.28, 3.00) | 0.89 |  | 0.84 (0.49, 1.43) | 0.52 |
|  | Females only | 4,001 (38,760) | 0.93 (0.71, 1.22) | 0.61 |  | 0.82 (0.48, 1.39) | 0.45 |  | 1.11 (0.84, 1.48) | 0.46 |
| Current GAD | All | 1,573 (80,861) | 1.36 (0.88, 2.09) | 0.16 |  | 1.10 (0.49, 2.45) | 0.82 |  | 1.28 (0.77, 2.12) | 0.35 |
|  | Males only | 542 (39,141) | 2.16 (1.00, 4.70) | 0.05 |  | 1.73 (0.46, 6.50) | 0.42 |  | 1.49 (0.52, 4.26) | 0.46 |
|  | Females only | 1,031 (41,720) | 1.09 (0.66, 1.81) | 0.73 |  | 0.86 (0.31, 2.38) | 0.77 |  | 1.20 (0.70, 2.07) | 0.50 |
| **Mental health outcome** | **Strata** | **N total** | **β (95% CI) per SD higher BMI** | **P** |  | **β (95% CI) per SD higher FA** | **P** |  | **β (95% CI) per SD higher UFA** | **P** |
| CIDI severity | All | 124,275 | 0.203 (0.071, 0.334) | 2.50E-03 |  | 0.07 (-0.181, 0.320) | 0.59 |  | 0.189 (0.029, 0.349) | 0.02 |
|  | Males only | 54,455 | 0.132 (-0.060, 0.325) | 0.18 |  | 0.002 (-0.343, 0.347) | 0.99 |  | 0.051 (-0.216, 0.318) | 0.71 |
|  | Females only | 69,820 | 0.252 (0.073, 0.432) | 5.90E-03 |  | 0.131 (-0.228, 0.490) | 0.47 |  | 0.269 (0.069, 0.468) | 8.20E-03 |
| PHQ9 severity | All | 124,275 | 0.461 (0.294, 0.629) | 6.50E-08 |  | 0.564 (0.245, 0.883) | 5.30E-04 |  | 0.532 (0.328, 0.735) | 3.00E-07 |
|  | Males only | 54,455 | 0.354 (0.107, 0.601) | 5.00E-03 |  | 0.472 (0.029, 0.915) | 0.04 |  | 0.258 (-0.084, 0.601) | 0.14 |
|  | Females only | 69,820 | 0.541 (0.314, 0.769) | 3.00E-06 |  | 0.131 (-0.228, 0.490) | 0.47 |  | 0.699 (0.447, 0.951) | 5.60E-08 |
| GAD severity | All | 123,774 | 0.097 (-0.057, 0.251) | 0.22 |  | 0.254 (-0.039, 0.547) | 0.09 |  | 0.047 (-0.139, 0.234) | 0.62 |
|  | Males only | 54,281 | 0.114 (-0.104, 0.331) | 0.31 |  | 0.307 (-0.082, 0.695) | 0.12 |  | -0.014 (-0.314, 0.287) | 0.93 |
|  | Females only | 69,493 | 0.084 (-0.131, 0.300) | 0.44 |  | 0.212 (-0.218, 0.642) | 0.33 |  | 0.085 (-0.154, 0.323) | 0.49 |
| Wellbeing score | All | 120,669 | -0.222 (-0.315, -0.129) | 2.90E-06 |  | -0.181 (-0.358, -0.003) | 0.05 |  | -0.171 (-0.283, -0.059) | 2.80E-03 |
|  | Males only | 52,736 | -0.154 (-0.297, -0.010) | 0.04 |  | 0.005 (-0.251, 0.262) | 0.97 |  | -0.006 (-0.205, 0.192) | 0.95 |
|  | Females only | 67,933 | -0.271 (-0.393, -0.148) | 1.50E-05 |  | -0.345 (-0.590, -0.099) | 5.90E-03 |  | -0.265 (-0.400, -0.130) | 1.20E-04 |

**Supplementary Table 4: 2-sample Mendelian randomization results for BMI in UK Biobank. Results from pleiotropy robust methods: Egger analysis, weighted median (WM) analysis and penalised weighted median analysis (PWM).**

|  |  |  |  |  |  | **BMI** |  |  |  |  |
| --- | --- | --- | --- | --- | --- | --- | --- | --- | --- | --- |
| **Trait** | **Strata** | **N cases (controls)** | **OR (95% CI) per SD higher BMI Egger** | **pEgger** | **egger_int** | **int_p** | **OR (95% CI) per SD higher BMI WM** | **pWM** | **OR (95% CI) per SD higher BMI PWM** | **pPWM** |
| Major depression | All | 34,739 (110,844) | 1.17 (0.90, 1.51) | 2.44E-01 | -0.00212 | 5.71E-01 | 1.07 (0.92, 1.23) | 3.69E-01 | 1.07 (0.93, 1.24) | 3.48E-01 |
|  | Males only | 10,808 (52,615) | 1.29 (0.89, 1.87) | 1.81E-01 | -0.00684 | 2.09E-01 | 1.12 (0.89, 1.40) | 3.33E-01 | 1.11 (0.88, 1.39) | 3.71E-01 |
|  | Females only | 23,931 (58,229) | 1.12 (0.82, 1.52) | 4.70E-01 | -0.00001 | 9.98E-01 | 1.04 (0.89, 1.23) | 5.98E-01 | 1.05 (0.88, 1.24) | 6.09E-01 |
| Severe major depression | All | 5,483 (140,100) | 1.25 (0.79, 1.96) | 3.41E-01 | 0.00051 | 9.38E-01 | 1.52 (1.14, 2.03) | 4.13E-03 | 1.55 (1.14, 2.12) | 5.21E-03 |
|  | Males only | 1,441 (61,982) | 2.49 (1.03, 6.03) | 4.76E-02 | -0.01842 | 1.56E-01 | 1.62 (0.96, 2.72) | 6.88E-02 | 1.68 (0.98, 2.87) | 5.70E-02 |
|  | Females only | 4,042 (78,118) | 0.97 (0.57, 1.62) | 8.93E-01 | 0.00757 | 3.17E-01 | 1.26 (0.88, 1.81) | 1.98E-01 | 1.27 (0.87, 1.86) | 2.14E-01 |
| Current depression | All | 2,641 (143,026) | 1.52 (0.79, 2.92) | 2.11E-01 | -0.00049 | 9.59E-01 | 1.16 (0.77, 1.76) | 4.71E-01 | 1.15 (0.75, 1.76) | 5.31E-01 |
|  | Males only | 962 (62,500) | 1.01 (0.36, 2.83) | 9.89E-01 | 0.00916 | 4.45E-01 | 1.18 (0.63, 2.19) | 6.09E-01 | 1.17 (0.62, 2.21) | 6.34E-01 |
|  | Females only | 1,679 (80,526) | 1.87 (0.79, 4.43) | 1.58E-01 | -0.00578 | 6.45E-01 | 1.68 (0.96, 2.92) | 6.74E-02 | 1.66 (0.94, 2.93) | 8.04E-02 |
| Severe current depression | All | 1,787 (143,880) | 1.43 (0.61, 3.33) | 4.16E-01 | 0.00748 | 5.46E-01 | 1.51 (0.92, 2.47) | 1.02E-01 | 1.29 (0.78, 2.13) | 3.23E-01 |
|  | Males only | 662 (62,800) | 1.10 (0.32, 3.83) | 8.82E-01 | 0.01732 | 2.71E-01 | 2.02 (0.89, 4.61) | 9.33E-02 | 2.00 (0.92, 4.33) | 7.89E-02 |
|  | Females only | 1,125 (81,080) | 1.69 (0.59, 4.83) | 3.29E-01 | 0.00107 | 9.44E-01 | 1.70 (0.86, 3.36) | 1.30E-01 | 1.70 (0.84, 3.43) | 1.41E-01 |
| GAD | All | 7,218 (89,440) | 1.41 (0.93, 2.15) | 1.14E-01 | -0.01105 | 7.49E-02 | 1.06 (0.82, 1.36) | 6.81E-01 | 1.05 (0.81, 1.37) | 6.95E-01 |
|  | Males only | 2,533 (43,739) | 1.04 (1.01, 1.08) | 2.71E-02 | -0.00093 | 6.37E-02 | 1.02 (0.99, 1.04) | 1.68E-01 | 1.01 (0.98, 1.04) | 3.73E-01 |
|  | Females only | 4,685 (45,701) | 1.12 (0.68, 1.85) | 6.48E-01 | -0.00770 | 2.91E-01 | 0.82 (0.59, 1.13) | 2.25E-01 | 0.81 (0.59, 1.13) | 2.14E-01 |
| Current GAD | All | 1,844 (94,798) | 1.41 (0.66, 3.00) | 3.74E-01 | -0.00463 | 6.63E-01 | 1.28 (0.78, 2.11) | 3.33E-01 | 1.28 (0.75, 2.17) | 3.62E-01 |
|  | Males only | 646 (45,622) | 0.98 (0.28, 3.44) | 9.74E-01 | 0.01630 | 3.34E-01 | 1.57 (0.69, 3.57) | 2.79E-01 | 1.53 (0.67, 3.46) | 3.12E-01 |
|  | Females only | 1,198 (49,176) | 1.66 (0.65, 4.25) | 2.95E-01 | -0.01527 | 2.42E-01 | 1.25 (0.66, 2.35) | 4.90E-01 | 1.23 (0.65, 2.33) | 5.26E-01 |
| **Trait** | **Strata** | **N total** | **β (95% CI) per SD higher BMI Egger** | **pEgger** | **egger_int** | **int_p** | **β (95% CI) per SD higher BMI WM** | **pWM** | **β (95% CI) per SD higher BMI PWM** | **pPWM** |
| CIDI severity | All | 145,668 | 0.214 (0.171) | 2.15E-01 | -0.00356 | 4.67E-01 | 0.117 (0.088) | 1.85E-01 | 0.117 (0.091) | 1.99E-01 |
|  | Males only | 63,462 | 0.205 (0.192) | 2.91E-01 | -0.00478 | 3.86E-01 | 0.158 (0.119) | 1.87E-01 | 0.155 (0.116) | 1.80E-01 |
|  | Females only | 82,206 | 0.230 (0.228) | 3.17E-01 | -0.00285 | 6.63E-01 | 0.132 (0.120) | 2.74E-01 | 0.130 (0.117) | 2.66E-01 |
| PHQ9 severity | All | 145,668 | 0.192 (0.222) | 3.92E-01 | 0.00510 | 4.23E-01 | 0.328 (0.114) | 4.16E-03 | 0.326 (0.113) | 3.97E-03 |
|  | Males only | 63,462 | -0.147 (0.263) | 5.76E-01 | 0.01224 | 1.06E-01 | 0.107 (0.149) | 4.74E-01 | 0.102 (0.152) | 5.02E-01 |
|  | Females only | 82,206 | 0.425 (0.288) | 1.45E-01 | 0.00022 | 9.79E-01 | 0.572 (0.147) | 9.82E-05 | 0.400 (0.146) | 6.03E-03 |
| GAD severity | All | 145,069 | 0.091 (0.185) | 6.25E-01 | -0.00155 | 7.70E-01 | 0.144 (0.106) | 1.76E-01 | 0.145 (0.103) | 1.58E-01 |
|  | Males only | 63,246 | -0.110 (0.203) | 5.89E-01 | 0.00573 | 3.25E-01 | 0.094 (0.137) | 4.92E-01 | 0.097 (0.133) | 4.67E-01 |
|  | Females only | 81,823 | 0.236 (0.251) | 3.50E-01 | -0.00709 | 3.24E-01 | 0.075 (0.136) | 5.81E-01 | 0.074 (0.137) | 5.88E-01 |
| Wellbeing score | All | 141,447 | -0.268 (0.139) | 5.79E-02 | 0.00362 | 3.64E-01 | -0.237 (0.063) | 1.76E-04 | -0.029 (0.066) | 6.58E-01 |
|  | Males only | 61,423 | -0.287 (0.176) | 1.08E-01 | 0.00633 | 2.12E-01 | -0.148 (0.093) | 1.10E-01 | -0.030 (0.095) | 7.57E-01 |
|  | Females only | 80,024 | -0.233 (0.167) | 1.66E-01 | 0.00091 | 8.48E-01 | -0.266 (0.085) | 1.71E-03 | -0.238 (0.093) | 1.05E-02 |

BMI=body mass index, FA= favourable adiposity, UFA=unfavourable adiposity. β represent standard deviation change in mental health outcome for standard deviation change in genetically determined adiposity trait, 95% confidence interval in brackets.

**Supplementary table 5: 1-sample MR BMI to mental health in all individuals and individuals not taking antidepressants**

| **Mental health outcome** | **Strata** | **N cases (controls)** | **OR (95% CI) per SD higher BMI** | **P** |  | **OR (95% CI) per SD higher favourable adiposity** | **P** |  | **OR (95% CI) per SD higher unfavourable adiposity** | **P** |
| --- | --- | --- | --- | --- | --- | --- | --- | --- | --- | --- |
| Major depression | All individuals | 29,594 (94,609) | 1.15 (1.03, 1.29) | 0.01 |  | 1.00 (0.81, 1.23) | 1.00 |  | 1.23 (1.08, 1.41) | 2.60E-03 |
|  | No antidepressants | 24,996 (91,846) | 1.14 (1.02, 1.29) | 0.03 |  | 0.97 (0.78, 1.22) | 0.80 |  | 1.23 (1.06, 1.42) | 5.70E-03 |
| Severe major depression | All individuals | 4,695 (119,508) | 1.30 (1.02, 1.66) | 0.04 |  | 1.17 (0.73, 1.86) | 0.51 |  | 1.37 (1.02, 1.85) | 0.04 |
|  | No antidepressants | 3,421 (113,421) | 1.35 (1.02, 1.80) | 0.04 |  | 0.99 (0.57, 1.69) | 0.96 |  | 1.35 (0.95, 1.92) | 0.09 |
| Current depression | All individuals | 2,238 (122,036) | 1.57 (1.11, 2.23) | 0.01 |  | 2.48 (1.27, 4.84) | 7.70E-03 |  | 2.10 (1.37, 3.21) | 6.40E-04 |
|  | No antidepressants | 1,542 (115,369) | 1.65 (1.08, 2.52) | 0.02 |  | 2.28 (1.03, 5.06) | 0.04 |  | 2.74 (1.64, 4.58) | 1.20E-04 |
| Severe current depression | All individuals | 1,514 (122,760) | 1.86 (1.22, 2.83) | 4.20E-03 |  | 2.57 (1.14, 5.76) | 0.02 |  | 2.73 (1.63, 4.57) | 1.30E-04 |
|  | No antidepressants | 1,010 (115,901) | 1.76 (1.05, 2.96) | 0.03 |  | 2.83 (1.06, 7.55) | 0.04 |  | 3.28 (1.74, 6.17) | 2.30E-04 |
| Atypical depression | All individuals | 2,508 (121,695) | 2.21 (1.59, 3.09) | 2.90E-06 |  | 2.23 (1.18, 4.21) | 0.01 |  | 2.28 (1.52, 3.42) | 6.80E-05 |
|  | No antidepressants | 1,883 (114,959) | 2.39 (1.62, 3.51) | 9.60E-06 |  | 2.86 (1.38, 5.92) | 4.60E-03 |  | 2.37 (1.48, 3.80) | 3.10E-04 |
| GAD | All individuals | 6,163 (76,284) | 1.08 (0.86, 1.35) | 0.51 |  | 1.07 (0.70, 1.62) | 0.76 |  | 1.02 (0.78, 1.33) | 0.89 |
|  | No antidepressants | 4,699 (74,457) | 1.12 (0.86, 1.44) | 0.40 |  | 0.84 (0.53, 1.35) | 0.47 |  | 1.12 (0.82, 1.51) | 0.48 |
| Current GAD | All individuals | 1,573 (80,861) | 1.36 (0.88, 2.09) | 0.16 |  | 1.10 (0.49, 2.45) | 0.82 |  | 1.28 (0.77, 2.12) | 0.35 |
|  | No antidepressants | 1,116 (78,028) | 1.55 (0.93, 2.58) | 0.09 |  | 0.69 (0.27, 1.77) | 0.44 |  | 1.63 (0.88, 2.99) | 0.12 |
| **Mental health outcome** | **Strata** | **N total** | **β (95% CI) per SD higher BMI** | **P** |  | **β (95% CI) per SD higher FA** | **P** |  | **β (95% CI) per SD higher UFA** | **P** |
| CIDI severity | All individuals | 124,275 | 0.203 (0.071, 0.334) | 2.50E-03 |  | 0.070 (-0.181, 0.320) | 0.59 |  | 0.189 (0.029, 0.349) | 0.02 |
|  | No antidepressants | 116,911 | 0.205 (0.071, 0.34) | 2.80E-03 |  | 0.049 (-0.205, 0.303) | 0.70 |  | 0.194 (0.030, 0.358) | 0.02 |
| PHQ9 severity | All individuals | 124,275 | 0.461 (0.294, 0.629) | 6.50E-08 |  | 0.564 (0.245, 0.883) | 5.30E-04 |  | 0.532 (0.328, 0.735) | 3.00E-07 |
|  | No antidepressants | 116,911 | 0.431 (0.269, 0.593) | 1.90E-07 |  | 0.513 (0.208, 0.819) | 1.00E-03 |  | 0.512 (0.315, 0.709) | 3.70E-07 |
| GAD severity | All individuals | 123,774 | 0.097 (-0.057, 0.251) | 0.22 |  | 0.254 (-0.039, 0.547) | 0.09 |  | 0.047 (-0.139, 0.234) | 0.62 |
|  | No antidepressants | 116,445 | 0.074 (-0.079, 0.226) | 0.34 |  | 0.198 (-0.088, 0.485) | 0.18 |  | 0.080 (-0.104, 0.265) | 0.39 |
| Wellbeing score | All individuals | 120,669 | -0.222 (-0.315, -0.129) | 2.90E-06 |  | -0.181 (-0.358, -0.003) | 0.05 |  | -0.171 (-0.283, -0.059) | 2.80E-03 |
|  | No antidepressants | 113,597 | -0.230 (-0.325, -0.136) | 1.60E-06 |  | -0.180 (-0.358, -0.002) | 0.05 |  | -0.154 (-0.268, -0.040) | 8.00E-03 |

**Supplementary Table 6: Mendelian randomization results for favourable adiposity in UK Biobank. Results from pleiotropy robust methods: Egger analysis, weighted median (WM) analysis and penalised weighted median analysis (PWM).**

|  |  |  |  |  | **FAVOURABLE ADIPOSITY** |  |  |  |  |  |
| --- | --- | --- | --- | --- | --- | --- | --- | --- | --- | --- |
| **Trait** | **Strata** | **N cases (controls)** | **OR (95% CI) per SD higher FA Egger** | **pEgger** | **egger_int** | **int_p** | **OR (95% CI) per SD higher FA WM** | **pWM** | **OR (95% CI) per SD higher FA PWM** | **pPWM** |
| Major depression | All | 34,739 (110,844) | 2.98 (1.28, 6.94) | 1.59E-02 | -0.01420 | 2.52E-02 | 1.60 (1.12, 2.28) | 9.00E-03 | 1.52 (1.07, 2.16) | 1.80E-02 |
|  | Males only | 10,808 (52,615) | 1.31 (0.31, 5.54) | 7.13E-01 | -0.00198 | 8.49E-01 | 1.17 (0.64, 2.16) | 6.08E-01 | 1.05 (0.57, 1.94) | 8.68E-01 |
|  | Females only | 23,931 (58,229) | 4.79 (1.73, 13.27) | 4.87E-03 | -0.02134 | 6.26E-03 | 1.04 (0.66, 1.62) | 8.72E-01 | 0.88 (0.53, 1.46) | 6.17E-01 |
| Severe major depression | All | 5,483 (140,100) | 1.80 (0.25, 13.08) | 5.67E-01 | -0.00342 | 8.12E-01 | 1.06 (0.50, 2.28) | 8.72E-01 | 1.03 (0.48, 2.24) | 9.35E-01 |
|  | Males only | 1,441 (61,982) | 0.29 (0.01, 5.87) | 4.24E-01 | 0.03097 | 1.00E-01 | 1.65 (0.43, 6.33) | 4.65E-01 | 1.65 (0.41, 6.71) | 4.84E-01 |
|  | Females only | 4,042 (78,118) | 3.76 (0.37, 38.58) | 2.73E-01 | -0.01664 | 3.27E-01 | 1.51 (0.61, 3.73) | 3.73E-01 | 1.35 (0.52, 3.46) | 5.38E-01 |
| Current depression | All | 2,641 (143,026) | 1.54 (0.16, 14.94) | 7.12E-01 | 0.00915 | 5.56E-01 | 4.41 (1.55, 12.54) | 5.39E-03 | 4.60 (1.58, 13.41) | 5.23E-03 |
|  | Males only | 962 (62,500) | 1.46 (0.03, 61.38) | 8.44E-01 | 0.02199 | 3.83E-01 | 4.24 (0.81, 22.13) | 8.68E-02 | 4.22 (0.73, 24.38) | 1.08E-01 |
|  | Females only | 1,679 (80,526) | 1.60 (0.09, 27.76) | 7.50E-01 | 0.00152 | 9.37E-01 | 2.40 (0.66, 8.66) | 1.82E-01 | 2.47 (0.69, 8.84) | 1.65E-01 |
| Severe current depression | All | 1,787 (143,880) | 1.95 (0.12, 30.67) | 6.39E-01 | 0.00669 | 6.56E-01 | 3.80 (1.13, 12.86) | 3.15E-02 | 3.34 (0.92, 12.05) | 6.58E-02 |
|  | Males only | 662 (62,800) | 2.16 (0.02, 197.62) | 7.41E-01 | 0.01744 | 5.18E-01 | 5.87 (0.78, 44.34) | 8.64E-02 | 5.82 (0.75, 45.36) | 9.28E-02 |
|  | Females only | 1,125 (81,080) | 1.81 (0.06, 58.78) | 7.42E-01 | 0.00050 | 9.80E-01 | 2.55 (0.52, 12.41) | 2.47E-01 | 2.55 (0.54, 12.12) | 2.40E-01 |
| GAD | All | 7,218 (89,440) | 2.31 (0.43, 12.48) | 3.39E-01 | -0.01096 | 3.72E-01 | 1.32 (0.64, 2.72) | 4.53E-01 | 1.29 (0.65, 2.58) | 4.66E-01 |
|  | Males only | 2,533 (43,739) | 1.00 (0.86, 1.15) | 9.57E-01 | 0.00054 | 6.07E-01 | 1.05 (0.99, 1.11) | 1.02E-01 | 1.05 (0.98, 1.13) | 1.94E-01 |
|  | Females only | 4,685 (45,701) | 4.39 (0.55, 35.05) | 1.72E-01 | -0.02379 | 1.20E-01 | 1.17 (0.49, 2.77) | 7.29E-01 | 0.84 (0.35, 2.04) | 7.05E-01 |
| Current GAD | All | 1,844 (94,798) | 0.90 (0.06, 13.78) | 9.41E-01 | -0.00078 | 9.68E-01 | 0.91 (0.26, 3.21) | 8.79E-01 | 0.86 (0.23, 3.15) | 8.19E-01 |
|  | Males only | 646 (45,622) | 1.49 (0.02, 141.57) | 8.66E-01 | -0.00103 | 9.73E-01 | 0.95 (0.12, 7.63) | 9.59E-01 | 0.87 (0.10, 7.28) | 8.99E-01 |
|  | Females only | 1,198 (49,176) | 0.51 (0.02, 15.28) | 7.00E-01 | 0.00237 | 9.13E-01 | 0.85 (0.17, 4.32) | 8.43E-01 | 0.84 (0.18, 3.89) | 8.21E-01 |
| **Trait** | **Strata** | **N total** | **β (95% CI) per SD higher FA Egger** | **pEgger** | **egger_int** | **int_p** | **β (95% CI) per SD higher FA WM** | **pWM** | **β (95% CI) per SD higher FA PWM** | **pPWM** |
| CIDI severity | All | 145,668 | 0.636 (0.462) | 1.78E-01 | -0.00735 | 2.67E-01 | 0.116 (0.208) | 5.78E-01 | 0.110 (0.217) | 6.12E-01 |
|  | Males only | 63,462 | -0.326 (0.731) | 6.59E-01 | 0.00410 | 6.93E-01 | -0.115 (0.293) | 6.95E-01 | -0.121 (0.295) | 6.81E-01 |
|  | Females only | 82,206 | 1.472 (0.614) | 2.22E-02 | -0.01750 | 5.08E-02 | 0.332 (0.311) | 2.86E-01 | 0.258 (0.308) | 4.02E-01 |
| PHQ9 severity | All | 145,668 | 0.485 (0.567) | 3.99E-01 | 0.00030 | 9.68E-01 | 0.624 (0.253) | 1.37E-02 | 0.757 (0.265) | 4.31E-03 |
|  | Males only | 63,462 | 0.667 (0.824) | 4.24E-01 | -0.00198 | 8.28E-01 | 0.356 (0.372) | 3.39E-01 | 0.367 (0.391) | 3.47E-01 |
|  | Females only | 82,206 | 0.322 (0.779) | 6.82E-01 | 0.00276 | 7.84E-01 | 0.651 (0.349) | 6.23E-02 | 0.646 (0.358) | 7.14E-02 |
| GAD severity | All | 145,069 | -0.077 (0.519) | 8.83E-01 | 0.00337 | 6.09E-01 | 0.126 (0.228) | 5.79E-01 | 0.139 (0.243) | 5.66E-01 |
|  | Males only | 63,246 | 0.075 (0.721) | 9.18E-01 | 0.00347 | 6.80E-01 | 0.374 (0.334) | 2.63E-01 | 0.366 (0.308) | 2.35E-01 |
|  | Females only | 81,823 | -0.198 (0.734) | 7.89E-01 | 0.00356 | 6.79E-01 | 0.104 (0.337) | 7.58E-01 | 0.105 (0.320) | 7.43E-01 |
| Wellbeing score | All | 141,447 | -0.068 (0.312) | 8.28E-01 | -0.00198 | 6.48E-01 | -0.221 (0.144) | 1.26E-01 | -0.260 (0.141) | 6.49E-02 |
|  | Males only | 61,423 | -0.289 (0.471) | 5.43E-01 | 0.00330 | 5.72E-01 | -0.072 (0.221) | 7.44E-01 | 0.058 (0.230) | 8.02E-01 |
|  | Females only | 80,024 | 0.146 (0.418) | 7.28E-01 | -0.00681 | 2.38E-01 | -0.204 (0.191) | 2.85E-01 | -0.081 (0.198) | 6.83E-01 |

BMI=body mass index, FA= favourable adiposity, UFA=unfavourable adiposity. β represent standard deviation change in mental health outcome for standard deviation change in genetically determined adiposity trait, 95% confidence interval in brackets.

**Supplementary Table 7: Mendelian randomization results for unfavourable adiposity in UK Biobank. Results from pleiotropy robust methods: Egger analysis, weighted median (WM) analysis and penalised weighted median analysis (PWM).**

|  |  |  |  | **UNFAVOURABLE ADIPOSITY** |  |  |  |  |  |  |
| --- | --- | --- | --- | --- | --- | --- | --- | --- | --- | --- |
| **Trait** | **Strata** | **N cases (controls)** | **OR (95% CI) per SD higher UFA Egger** | **pEgger** | **egger_int** | **int_p** | **OR (95% CI) per SD higher UFA WM** | **pWM** | **OR (95% CI) per SD higher UFA PWM** | **pPWM** |
| Major depression | All | 34,739 (110,844) | 1.80 (0.96, 3.34) | 7.31E-02 | -0.00632 | 3.41E-01 | 1.15 (0.92, 1.44) | 2.20E-01 | 1.15 (0.91, 1.45) | 2.43E-01 |
|  | Males only | 10,808 (52,615) | 2.11 (0.74, 6.04) | 1.71E-01 | -0.00875 | 4.34E-01 | 1.54 (1.04, 2.29) | 3.25E-02 | 1.55 (1.01, 2.40) | 4.59E-02 |
|  | Females only | 23,931 (58,229) | 1.73 (0.90, 3.33) | 1.10E-01 | -0.00586 | 4.01E-01 | 1.19 (0.90, 1.58) | 2.16E-01 | 1.18 (0.90, 1.57) | 2.36E-01 |
| Severe major depression | All | 5,483 (140,100) | 3.39 (1.05, 10.90) | 4.81E-02 | -0.01647 | 1.17E-01 | 1.59 (0.97, 2.60) | 6.54E-02 | 1.39 (0.83, 2.33) | 2.10E-01 |
|  | Males only | 1,441 (61,982) | 7.65 (0.84, 69.97) | 7.98E-02 | -0.02964 | 1.82E-01 | 1.61 (0.65, 4.01) | 3.03E-01 | 1.30 (0.51, 3.32) | 5.80E-01 |
|  | Females only | 4,042 (78,118) | 2.54 (0.64, 10.04) | 1.92E-01 | -0.01160 | 3.88E-01 | 1.58 (0.86, 2.91) | 1.41E-01 | 1.57 (0.85, 2.89) | 1.46E-01 |
| Current depression | All | 2,641 (143,026) | 1.25 (0.24, 6.65) | 7.94E-01 | 0.01570 | 3.78E-01 | 2.98 (1.43, 6.20) | 3.45E-03 | 1.97 (0.96, 4.05) | 6.29E-02 |
|  | Males only | 962 (62,500) | 0.83 (0.05, 12.94) | 8.96E-01 | 0.01410 | 5.85E-01 | 1.17 (0.38, 3.57) | 7.88E-01 | 1.16 (0.36, 3.71) | 7.99E-01 |
|  | Females only | 1,679 (80,526) | 1.52 (0.15, 15.84) | 7.30E-01 | 0.01697 | 4.97E-01 | 3.84 (1.45, 10.20) | 6.87E-03 | 3.68 (1.36, 9.94) | 1.02E-02 |
| Severe current depression | All | 1,787 (143,880) | 0.63 (0.08, 5.04) | 6.67E-01 | 0.03646 | 1.05E-01 | 2.06 (0.83, 5.14) | 1.20E-01 | 2.03 (0.86, 4.78) | 1.07E-01 |
|  | Males only | 662 (62,800) | 0.36 (0.01, 10.02) | 5.53E-01 | 0.04043 | 2.56E-01 | 1.08 (0.27, 4.43) | 9.11E-01 | 1.04 (0.25, 4.37) | 9.54E-01 |
|  | Females only | 1,125 (81,080) | 0.91 (0.07, 12.34) | 9.43E-01 | 0.03347 | 2.31E-01 | 2.96 (0.88, 9.90) | 7.84E-02 | 3.10 (0.91, 10.56) | 6.98E-02 |
| GAD | All | 7,218 (89,440) | 1.87 (0.65, 5.37) | 2.55E-01 | -0.01228 | 2.78E-01 | 1.09 (0.69, 1.72) | 7.08E-01 | 1.11 (0.71, 1.73) | 6.49E-01 |
|  | Males only | 2,533 (43,739) | 1.08 (0.98, 1.19) | 1.13E-01 | -0.00165 | 1.14E-01 | 1.04 (1.00, 1.08) | 7.71E-02 | 1.03 (0.98, 1.08) | 2.74E-01 |
|  | Females only | 4,685 (45,701) | 1.06 (0.29, 3.91) | 9.30E-01 | 0.00033 | 9.80E-01 | 0.80 (0.46, 1.40) | 4.41E-01 | 0.81 (0.47, 1.41) | 4.57E-01 |
| Current GAD | All | 1,844 (94,798) | 4.45 (0.60, 32.99) | 1.53E-01 | -0.02356 | 2.55E-01 | 1.56 (0.64, 3.81) | 3.33E-01 | 1.55 (0.65, 3.70) | 3.21E-01 |
|  | Males only | 646 (45,622) | 3.61 (0.13, 101.82) | 4.56E-01 | -0.01762 | 5.89E-01 | 1.14 (0.29, 4.46) | 8.45E-01 | 1.14 (0.27, 4.78) | 8.55E-01 |
|  | Females only | 1,198 (49,176) | 4.50 (0.35, 57.20) | 2.54E-01 | -0.02500 | 3.56E-01 | 1.42 (0.48, 4.22) | 5.25E-01 | 1.37 (0.45, 4.22) | 5.81E-01 |
| **Trait** | **Strata** | **N total** | **β (95% CI) per SD higher UFA Egger** | **pEgger** | **egger_int** | **int_p** | **β (95% CI) per SD higher UFA WM** | **pWM** | **β (95% CI) per SD higher UFA PWM** | **pPWM** |
| CIDI severity | All | 145,668 | 0.600 (0.379) | 1.22E-01 | -0.00759 | 3.38E-01 | 0.237 (0.152) | 1.18E-01 | 0.235 (0.150) | 1.18E-01 |
|  | Males only | 63,462 | 0.771 (0.449) | 9.49E-02 | -0.00873 | 3.27E-01 | 0.262 (0.194) | 1.76E-01 | 0.262 (0.198) | 1.86E-01 |
|  | Females only | 82,206 | 0.470 (0.528) | 3.80E-01 | -0.00796 | 4.71E-01 | 0.215 (0.210) | 3.04E-01 | 0.212 (0.216) | 3.26E-01 |
| PHQ9 severity | All | 145,668 | 0.591 (0.499) | 2.44E-01 | -0.00053 | 9.59E-01 | 0.643 (0.183) | 4.28E-04 | 0.639 (0.186) | 5.79E-04 |
|  | Males only | 63,462 | 0.614 (0.604) | 3.16E-01 | 0.00541 | 6.67E-01 | 1.058 (0.254) | 3.05E-05 | 1.048 (0.254) | 3.57E-05 |
|  | Females only | 82,206 | 0.471 (0.704) | 5.08E-01 | -0.00651 | 6.57E-01 | 0.087 (0.269) | 7.46E-01 | 0.107 (0.261) | 6.81E-01 |
| GAD severity | All | 145,069 | 0.077 (0.435) | 8.61E-01 | -0.00161 | 8.59E-01 | 0.087 (0.175) | 6.17E-01 | 0.281 (0.178) | 1.14E-01 |
|  | Males only | 63,246 | -0.063 (0.567) | 9.12E-01 | 0.00228 | 8.47E-01 | 0.148 (0.234) | 5.26E-01 | 0.230 (0.230) | 3.17E-01 |
|  | Females only | 81,823 | 0.211 (0.577) | 7.17E-01 | -0.00607 | 6.14E-01 | -0.055 (0.230) | 8.10E-01 | -0.053 (0.253) | 8.34E-01 |
| WellbeingScore | All | 141,447 | -0.505 (0.344) | 1.50E-01 | 0.00675 | 3.48E-01 | -0.312 (0.109) | 4.33E-03 | -0.073 (0.122) | 5.49E-01 |
|  | Males only | 61,423 | -0.377 (0.339) | 2.74E-01 | 0.00060 | 9.33E-01 | -0.425 (0.138) | 2.11E-03 | -0.322 (0.150) | 3.15E-02 |
|  | Females only | 80,024 | -0.610 (0.504) | 2.34E-01 | 0.01353 | 2.02E-01 | -0.165 (0.162) | 3.09E-01 | -0.230 (0.169) | 1.73E-01 |

BMI=body mass index, FA= favourable adiposity, UFA=unfavourable adiposity. β represent standard deviation change in mental health outcome for standard deviation change in genetically determined adiposity trait, 95% confidence interval in brackets.

**Supplementary table 8: Summary of the results from the non-linear Mendelian randomisation for all mental health outcomes against BMI**

|  |  | **Evidence of non-linearity from piecewise linear model** | |
| --- | --- | --- | --- |
| **Mental health outcome** | **Strata** | **Quadratic p value** | **Cochran Q p value** |
| Major depression | All | 0.222 | 0.357 |
| Major depression | Males only | 0.164 | 0.127 |
| Major depression | Females only | 0.647 | 0.441 |
| Severe major depression | All | 0.786 | 0.599 |
| Severe major depression | Males only | 0.795 | 0.967 |
| Severe major depression | Females only | 0.775 | 0.312 |
| Current depression | All | 0.081 | 0.12 |
| Current depression | Males only | 0.485 | 0.435 |
| Current depression | Females only | 0.068 | 0.381 |
| Severe current depression | All | 0.188 | 0.147 |
| Severe current depression | Males only | 0.334 | 0.677 |
| Severe current depression | Females only | 0.232 | 0.531 |
| GAD | All | 0.139 | 0.256 |
| GAD | Males only | 0.592 | 0.943 |
| GAD | Females only | 0.162 | 0.192 |
| Current GAD | All | 0.003 | 0.024 |
| Current GAD | Males only | 0.631 | 0.81 |
| Current GAD | Females only | 0.002 | 0.053 |
| CIDI severity | All | 0.575 | 0.35 |
| CIDI severity | Males only | 0.039 | 0.098 |
| CIDI severity | Females only | 0.24 | 0.108 |
| PHQ9 severity | All | 2.21E-03 | 0.023 |
| PHQ9 severity | Males only | 6.28E-04 | 0.028 |
| PHQ9 severity | Females only | 0.199 | 0.826 |
| GAD severity | All | 0.105 | 0.596 |
| GAD severity | Males only | 0.02 | 0.215 |
| GAD severity | Females only | 0.502 | 0.569 |
| Wellbeing score | All | 9.33E-08 | 1.60E-05 |
| Wellbeing score | Males only | 4.46E-05 | 0.011 |
| Wellbeing score | Females only | 5.17E-04 | 0.015 |

**Supplementary table 9: List of variants used as instruments for 2-sample MR and for creation of the genetic risk scores**

| **Test trait** | **SNP** | **Trait raising allele** | **Other allele** | **Beta** | **SE** | **SNP source** |
| --- | --- | --- | --- | --- | --- | --- |
| Favourable adiposity | rs10876529 | C | T | 0.0105 | 0.0015 | Martin et al. Unpublished |
| Favourable adiposity | rs11045172 | C | A | 0.0110 | 0.0019 | Martin et al. Unpublished |
| Favourable adiposity | rs11135038 | G | T | 0.0139 | 0.0017 | Martin et al. Unpublished |
| Favourable adiposity | rs113222038 | C | T | 0.0113 | 0.0017 | Martin et al. Unpublished |
| Favourable adiposity | rs11664106 | T | A | 0.0093 | 0.0016 | Martin et al. Unpublished |
| Favourable adiposity | rs12130231 | A | G | 0.0204 | 0.0015 | Martin et al. Unpublished |
| Favourable adiposity | rs12369179 | C | T | 0.0293 | 0.0026 | Martin et al. Unpublished |
| Favourable adiposity | rs12441543 | A | G | 0.0115 | 0.0016 | Martin et al. Unpublished |
| Favourable adiposity | rs12681990 | T | C | 0.0119 | 0.0020 | Martin et al. Unpublished |
| Favourable adiposity | rs12940684 | C | T | 0.0113 | 0.0016 | Martin et al. Unpublished |
| Favourable adiposity | rs13132853 | A | G | 0.0089 | 0.0016 | Martin et al. Unpublished |
| Favourable adiposity | rs13389219 | T | C | 0.0169 | 0.0015 | Martin et al. Unpublished |
| Favourable adiposity | rs142186653 | C | A | 0.0125 | 0.0018 | Martin et al. Unpublished |
| Favourable adiposity | rs2802774 | A | C | 0.0108 | 0.0015 | Martin et al. Unpublished |
| Favourable adiposity | rs2943653 | C | T | 0.0158 | 0.0016 | Martin et al. Unpublished |
| Favourable adiposity | rs30351 | G | A | 0.0119 | 0.0017 | Martin et al. Unpublished |
| Favourable adiposity | rs4450871 | G | A | 0.0086 | 0.0015 | Martin et al. Unpublished |
| Favourable adiposity | rs4684847 | T | C | 0.0289 | 0.0023 | Martin et al. Unpublished |
| Favourable adiposity | rs4821764 | G | A | 0.0169 | 0.0015 | Martin et al. Unpublished |
| Favourable adiposity | rs4976033 | A | G | 0.0116 | 0.0015 | Martin et al. Unpublished |
| Favourable adiposity | rs555162510 | A | AT | 0.0241 | 0.0020 | Martin et al. Unpublished |
| Favourable adiposity | rs573454216 | A | G | 0.0089 | 0.0015 | Martin et al. Unpublished |
| Favourable adiposity | rs6029180 | G | A | 0.0090 | 0.0016 | Martin et al. Unpublished |
| Favourable adiposity | rs62271373 | T | A | 0.0240 | 0.0032 | Martin et al. Unpublished |
| Favourable adiposity | rs6977416 | G | A | 0.0118 | 0.0016 | Martin et al. Unpublished |
| Favourable adiposity | rs7133378 | A | G | 0.0189 | 0.0016 | Martin et al. Unpublished |
| Favourable adiposity | rs7233512 | G | A | 0.0107 | 0.0016 | Martin et al. Unpublished |
| Favourable adiposity | rs7258937 | T | C | 0.0158 | 0.0015 | Martin et al. Unpublished |
| Favourable adiposity | rs72697297 | T | C | 0.0150 | 0.0020 | Martin et al. Unpublished |
| Favourable adiposity | rs72959041 | G | A | 0.0233 | 0.0035 | Martin et al. Unpublished |
| Favourable adiposity | rs972283 | A | G | 0.0144 | 0.0015 | Martin et al. Unpublished |
| Favourable adiposity | rs9764678 | C | T | 0.0105 | 0.0017 | Martin et al. Unpublished |
| Favourable adiposity | rs9851766 | A | G | 0.0137 | 0.0020 | Martin et al. Unpublished |
| Favourable adiposity | rs987469 | C | G | 0.0123 | 0.0015 | Martin et al. Unpublished |
| Favourable adiposity | rs998584 | C | A | 0.0092 | 0.0015 | Martin et al. Unpublished |
| Favourable adiposity | rs2980888 | C | T | 0.0121 | 0.0016 | Martin et al. Unpublished |
| Unfavourable adiposity | 1:113202203_TCTCTC_T | TCTCTC | T | 0.0141 | 0.0019 | Martin et al. Unpublished |
| Unfavourable adiposity | 1:72767554_CA_C | CA | C | 0.0199 | 0.0019 | Martin et al. Unpublished |
| Unfavourable adiposity | 14:79940130_TAGGAGTTTTTCCAGATCATTAGCCACTTATACGGAG_T | T | TAGGAGTTTTTCCAGATCATTAGCCACTTATACGGAG | 0.0216 | 0.0019 | Martin et al. Unpublished |
| Unfavourable adiposity | 15:73322940_AT_A | A | AT | 0.0144 | 0.0015 | Martin et al. Unpublished |
| Unfavourable adiposity | 5:87969925_CGG_C | C | CGG | 0.0247 | 0.0022 | Martin et al. Unpublished |
| Unfavourable adiposity | 6:34650934_CGT_C | C | CGT | 0.0256 | 0.0021 | Martin et al. Unpublished |
| Unfavourable adiposity | rs10623997 | T | TATAATA | 0.0148 | 0.0018 | Martin et al. Unpublished |
| Unfavourable adiposity | rs10756713 | A | G | 0.0193 | 0.0015 | Martin et al. Unpublished |
| Unfavourable adiposity | rs10938397 | G | A | 0.0199 | 0.0015 | Martin et al. Unpublished |
| Unfavourable adiposity | rs11122450 | T | G | 0.0096 | 0.0015 | Martin et al. Unpublished |
| Unfavourable adiposity | rs11642015 | T | C | 0.0413 | 0.0015 | Martin et al. Unpublished |
| Unfavourable adiposity | rs11666808 | T | C | 0.0167 | 0.0015 | Martin et al. Unpublished |
| Unfavourable adiposity | rs13107325 | T | C | 0.0306 | 0.0028 | Martin et al. Unpublished |
| Unfavourable adiposity | rs143684747 | AC | A | 0.0288 | 0.0020 | Martin et al. Unpublished |
| Unfavourable adiposity | rs1471740 | C | T | 0.0097 | 0.0017 | Martin et al. Unpublished |
| Unfavourable adiposity | rs17764730 | C | T | 0.0117 | 0.0017 | Martin et al. Unpublished |
| Unfavourable adiposity | rs2112347 | T | G | 0.0179 | 0.0015 | Martin et al. Unpublished |
| Unfavourable adiposity | rs2274224 | G | C | 0.0166 | 0.0015 | Martin et al. Unpublished |
| Unfavourable adiposity | rs236660 | C | T | 0.0141 | 0.0016 | Martin et al. Unpublished |
| Unfavourable adiposity | rs3764002 | C | T | 0.0189 | 0.0017 | Martin et al. Unpublished |
| Unfavourable adiposity | rs4755725 | C | A | 0.0167 | 0.0016 | Martin et al. Unpublished |
| Unfavourable adiposity | rs4776985 | T | G | 0.0201 | 0.0018 | Martin et al. Unpublished |
| Unfavourable adiposity | rs4790292 | C | A | 0.0209 | 0.0021 | Martin et al. Unpublished |
| Unfavourable adiposity | rs4876611 | G | A | 0.0179 | 0.0017 | Martin et al. Unpublished |
| Unfavourable adiposity | rs539515 | C | A | 0.0282 | 0.0018 | Martin et al. Unpublished |
| Unfavourable adiposity | rs55931203 | T | C | 0.0210 | 0.0019 | Martin et al. Unpublished |
| Unfavourable adiposity | rs56186137 | G | A | 0.0225 | 0.0015 | Martin et al. Unpublished |
| Unfavourable adiposity | rs61888762 | G | C | 0.0177 | 0.0016 | Martin et al. Unpublished |
| Unfavourable adiposity | rs6567160 | C | T | 0.0256 | 0.0018 | Martin et al. Unpublished |
| Unfavourable adiposity | rs6602997 | T | C | 0.0223 | 0.0016 | Martin et al. Unpublished |
| Unfavourable adiposity | rs6752378 | A | C | 0.0230 | 0.0015 | Martin et al. Unpublished |
| Unfavourable adiposity | rs7124681 | A | C | 0.0224 | 0.0015 | Martin et al. Unpublished |
| Unfavourable adiposity | rs7132908 | A | G | 0.0191 | 0.0015 | Martin et al. Unpublished |
| Unfavourable adiposity | rs71658797 | A | T | 0.0237 | 0.0023 | Martin et al. Unpublished |
| Unfavourable adiposity | rs72892910 | T | G | 0.0233 | 0.0020 | Martin et al. Unpublished |
| Unfavourable adiposity | rs771025058 | AAG | A | 0.0171 | 0.0015 | Martin et al. Unpublished |
| Unfavourable adiposity | rs8049669 | A | T | 0.0159 | 0.0015 | Martin et al. Unpublished |
| Unfavourable adiposity | rs9358912 | G | T | 0.0216 | 0.0017 | Martin et al. Unpublished |
| BMI | rs1000940 | G | A | 0.0192 | 0.0034 | Locke et al. PMID: 25673413 |
| BMI | rs10132280 | C | A | 0.0230 | 0.0034 | Locke et al. PMID: 25673413 |
| BMI | rs1016287 | T | C | 0.0229 | 0.0034 | Locke et al. PMID: 25673413 |
| BMI | rs10182181 | G | A | 0.0307 | 0.0031 | Locke et al. PMID: 25673413 |
| BMI | rs10733682 | A | G | 0.0174 | 0.0031 | Locke et al. PMID: 25673413 |
| BMI | rs10938397 | G | A | 0.0402 | 0.0031 | Locke et al. PMID: 25673413 |
| BMI | rs10968576 | G | A | 0.0249 | 0.0033 | Locke et al. PMID: 25673413 |
| BMI | rs11057405 | G | A | 0.0307 | 0.0055 | Locke et al. PMID: 25673413 |
| BMI | rs11126666 | A | G | 0.0207 | 0.0034 | Locke et al. PMID: 25673413 |
| BMI | rs11165643 | T | C | 0.0218 | 0.0031 | Locke et al. PMID: 25673413 |
| BMI | rs11191560 | C | T | 0.0308 | 0.0053 | Locke et al. PMID: 25673413 |
| BMI | rs11583200 | C | T | 0.0177 | 0.0031 | Locke et al. PMID: 25673413 |
| BMI | rs1167827 | G | A | 0.0202 | 0.0033 | Locke et al. PMID: 25673413 |
| BMI | rs11688816 | G | A | 0.0172 | 0.0031 | Locke et al. PMID: 25673413 |
| BMI | rs11727676 | T | C | 0.0358 | 0.0064 | Locke et al. PMID: 25673413 |
| BMI | rs11847697 | T | C | 0.0492 | 0.0084 | Locke et al. PMID: 25673413 |
| BMI | rs12286929 | G | A | 0.0217 | 0.0031 | Locke et al. PMID: 25673413 |
| BMI | rs12401738 | A | G | 0.0211 | 0.0033 | Locke et al. PMID: 25673413 |
| BMI | rs12429545 | A | G | 0.0334 | 0.0047 | Locke et al. PMID: 25673413 |
| BMI | rs12446632 | G | A | 0.0403 | 0.0046 | Locke et al. PMID: 25673413 |
| BMI | rs12566985 | G | A | 0.0242 | 0.0031 | Locke et al. PMID: 25673413 |
| BMI | rs12885454 | C | A | 0.0207 | 0.0033 | Locke et al. PMID: 25673413 |
| BMI | rs12940622 | G | A | 0.0182 | 0.0031 | Locke et al. PMID: 25673413 |
| BMI | rs13021737 | G | A | 0.0601 | 0.0040 | Locke et al. PMID: 25673413 |
| BMI | rs13078960 | G | T | 0.0297 | 0.0039 | Locke et al. PMID: 25673413 |
| BMI | rs13191362 | A | G | 0.0277 | 0.0048 | Locke et al. PMID: 25673413 |
| BMI | rs1516725 | C | T | 0.0451 | 0.0046 | Locke et al. PMID: 25673413 |
| BMI | rs1528435 | T | C | 0.0178 | 0.0031 | Locke et al. PMID: 25673413 |
| BMI | rs1558902 | A | T | 0.0818 | 0.0031 | Locke et al. PMID: 25673413 |
| BMI | rs16851483 | T | G | 0.0483 | 0.0077 | Locke et al. PMID: 25673413 |
| BMI | rs16951275 | T | C | 0.0311 | 0.0037 | Locke et al. PMID: 25673413 |
| BMI | rs17001654 | G | C | 0.0306 | 0.0053 | Locke et al. PMID: 25673413 |
| BMI | rs17024393 | C | T | 0.0658 | 0.0088 | Locke et al. PMID: 25673413 |
| BMI | rs17094222 | C | T | 0.0249 | 0.0038 | Locke et al. PMID: 25673413 |
| BMI | rs17405819 | T | C | 0.0224 | 0.0033 | Locke et al. PMID: 25673413 |
| BMI | rs17724992 | A | G | 0.0194 | 0.0035 | Locke et al. PMID: 25673413 |
| BMI | rs1808579 | C | T | 0.0167 | 0.0031 | Locke et al. PMID: 25673413 |
| BMI | rs1928295 | T | C | 0.0188 | 0.0031 | Locke et al. PMID: 25673413 |
| BMI | rs2033529 | G | A | 0.0190 | 0.0033 | Locke et al. PMID: 25673413 |
| BMI | rs2033732 | C | T | 0.0192 | 0.0035 | Locke et al. PMID: 25673413 |
| BMI | rs205262 | G | A | 0.0221 | 0.0035 | Locke et al. PMID: 25673413 |
| BMI | rs2075650 | A | G | 0.0258 | 0.0045 | Locke et al. PMID: 25673413 |
| BMI | rs2112347 | T | G | 0.0261 | 0.0031 | Locke et al. PMID: 25673413 |
| BMI | rs2121279 | T | C | 0.0245 | 0.0044 | Locke et al. PMID: 25673413 |
| BMI | rs2176598 | T | C | 0.0198 | 0.0036 | Locke et al. PMID: 25673413 |
| BMI | rs2207139 | G | A | 0.0447 | 0.0040 | Locke et al. PMID: 25673413 |
| BMI | rs2245368 | C | T | 0.0317 | 0.0057 | Locke et al. PMID: 25673413 |
| BMI | rs2287019 | C | T | 0.0360 | 0.0042 | Locke et al. PMID: 25673413 |
| BMI | rs2365389 | C | T | 0.0200 | 0.0031 | Locke et al. PMID: 25673413 |
| BMI | rs2650492 | A | G | 0.0207 | 0.0035 | Locke et al. PMID: 25673413 |
| BMI | rs2820292 | C | A | 0.0195 | 0.0031 | Locke et al. PMID: 25673413 |
| BMI | rs29941 | G | A | 0.0182 | 0.0033 | Locke et al. PMID: 25673413 |
| BMI | rs3101336 | C | T | 0.0334 | 0.0031 | Locke et al. PMID: 25673413 |
| BMI | rs3736485 | A | G | 0.0176 | 0.0031 | Locke et al. PMID: 25673413 |
| BMI | rs3810291 | A | G | 0.0283 | 0.0036 | Locke et al. PMID: 25673413 |
| BMI | rs3817334 | T | C | 0.0262 | 0.0031 | Locke et al. PMID: 25673413 |
| BMI | rs3849570 | A | C | 0.0188 | 0.0034 | Locke et al. PMID: 25673413 |
| BMI | rs4256980 | G | C | 0.0209 | 0.0031 | Locke et al. PMID: 25673413 |
| BMI | rs4740619 | T | C | 0.0179 | 0.0031 | Locke et al. PMID: 25673413 |
| BMI | rs543874 | G | A | 0.0482 | 0.0039 | Locke et al. PMID: 25673413 |
| BMI | rs6477694 | C | T | 0.0174 | 0.0031 | Locke et al. PMID: 25673413 |
| BMI | rs6567160 | C | T | 0.0556 | 0.0036 | Locke et al. PMID: 25673413 |
| BMI | rs657452 | A | G | 0.0227 | 0.0031 | Locke et al. PMID: 25673413 |
| BMI | rs6804842 | G | A | 0.0185 | 0.0031 | Locke et al. PMID: 25673413 |
| BMI | rs7138803 | A | G | 0.0315 | 0.0031 | Locke et al. PMID: 25673413 |
| BMI | rs7141420 | T | C | 0.0235 | 0.0031 | Locke et al. PMID: 25673413 |
| BMI | rs7243357 | T | G | 0.0217 | 0.0040 | Locke et al. PMID: 25673413 |
| BMI | rs758747 | T | C | 0.0225 | 0.0037 | Locke et al. PMID: 25673413 |
| BMI | rs7599312 | G | A | 0.0220 | 0.0034 | Locke et al. PMID: 25673413 |
| BMI | rs7899106 | G | A | 0.0395 | 0.0071 | Locke et al. PMID: 25673413 |
| BMI | rs9581854 | T | C | 0.0300 | 0.0050 | Locke et al. PMID: 25673413 |
| BMI | rs9400239 | C | T | 0.0188 | 0.0033 | Locke et al. PMID: 25673413 |
| BMI | rs9925964 | A | G | 0.0192 | 0.0031 | Locke et al. PMID: 25673413 |

**Supplementary Table 10:** **The association between BMI/FA/UFA GRS and BMI/BFP by sex in the UK Biobank.**

| **Sex** | **Exposure** | **Outcome** | **beta** | **se** | **P** | **F** | **R**^2^ |
| --- | --- | --- | --- | --- | --- | --- | --- |
| All | BMI GRS | BMI | 0.024 | 3.01E-04 | 0.00E+00 | 6194.7 | 0.0161 |
| Males only | BMI GRS | BMI | 0.023 | 4.05E-04 | 0.00E+00 | 3234.21 | 0.0182 |
| Females only | BMI GRS | BMI | 0.024 | 4.36E-04 | 0.00E+00 | 3056.64 | 0.0148 |
| All | FA GRS | BFP | 0.018 | 4.36E-04 | 0.00E+00 | 1734.34 | 0.0046 |
| Males only | FA GRS | BFP | 0.018 | 5.82E-04 | 2.00E-213 | 975.14 | 0.0057 |
| Females only | FA GRS | BFP | 0.018 | 6.36E-04 | 6.00E-178 | 810.6 | 0.0040 |
| All | FA GRS | BMI | 0.009 | 4.33E-04 | 1.20E-93 | 421.63 | 0.0011 |
| Males only | FA GRS | BMI | 0.008 | 5.84E-04 | 1.40E-41 | 182.57 | 0.0010 |
| Females only | FA GRS | BMI | 0.010 | 6.30E-04 | 1.20E-53 | 237.88 | 0.0012 |
| All | UFA GRS | BFP | 0.027 | 4.26E-04 | 0.00E+00 | 3961.08 | 0.0105 |
| Males only | UFA GRS | BFP | 0.025 | 5.70E-04 | 0.00E+00 | 1859.84 | 0.0107 |
| Females only | UFA GRS | BFP | 0.029 | 6.20E-04 | 0.00E+00 | 2132.71 | 0.0105 |
| All | UFA GRS | BMI | 0.025 | 4.23E-04 | 0.00E+00 | 3505.39 | 0.0092 |
| Males only | UFA GRS | BMI | 0.023 | 5.71E-04 | 0.00E+00 | 1642.15 | 0.0093 |
| Females only | UFA GRS | BMI | 0.027 | 6.13E-04 | 0.00E+00 | 1880.26 | 0.0091 |

**Supplementary table 11: Antidepressant drug names, codes from UK Biobank, field 20003, http://biobank.ndph.ox.ac.uk/showcase/coding.cgi?id=4&nl=1 and antidepressant class.**

| **Antidepressant** | **UK Biobank code** | **Class** |
| --- | --- | --- |
| faverin | 1140867860 | SSRI |
| prozac | 1140867876 | SSRI |
| sertraline | 1140867878 | SSRI |
| lustral | 1140867884 | SSRI |
| paroxetine | 1140867888 | SSRI |
| fluoxetine | 1140879540 | SSRI |
| fluvoxamine | 1140879544 | SSRI |
| seroxat | 1140882236 | SSRI |
| citalopram | 1140921600 | SSRI |
| cipramil | 1141151946 | SSRI |
| escitalopram | 1141180212 | SSRI |
| cipralex | 1141190158 | SSRI |
| prothiaden | 1140867624 | TCA |
| doxepin | 1140867640 | TCA |
| tryptizol | 1140867668 | TCA |
| anafranil | 1140867690 | TCA |
| lofepramine | 1140867726 | TCA |
| trimipramine | 1140867756 | TCA |
| surmontil | 1140867758 | TCA |
| ludiomil | 1140867784 | TCA |
| norval | 1140867812 | TCA |
| nortriptyline | 1140867818 | TCA |
| triptafen | 1140867934 | TCA |
| amitriptyline + chlordiazepoxide | 1140867938 | TCA |
| fluphenazine hydrochloride+nortriptyline | 1140867940 | TCA |
| amitriptyline hydrochloride+perphenazine | 1140867948 | TCA |
| amitriptyline | 1140879616 | TCA |
| clomipramine | 1140879620 | TCA |
| dothiepin | 1140879628 | TCA |
| imipramine | 1140879630 | TCA |
| sinequan | 1140882312 | TCA |
| dosulepin | 1140909806 | TCA |
| allegron | 1140867820 | TCA |
| fluphenazine hcl+nortriptyline | 1140867942 | TCA |
| elavil | 1140867658 | TCA |
| maproptiline | 1140879552 | TCA |
| tofranil | 1140867712 | TCA |
| desipramine | 1140879624 | TCA |
| protriptyline | 1140879632 | TCA |
| butriptyline | 1140856074 | TCA |
| evadyne | 1140856076 | TCA |
| praminil | 1140856144 | TCA |
| lomont | 1141146062 | TCA |
| gamanil | 1140882310 | TCA |
| aventyl | 1140867824 | TCA |
| amoxapine | 1140867774 | TCA |
| iprindole | 1140867720 | TCA |
| prondol | 1140867722 | TCA |
| phenelzine | 1140867850 | MAOI |
| nardil | 1140867852 | MAOI |
| isocarboxazid | 1140867856 | MAOI |
| tranylcypromine | 1140867914 | MAOI |
| moclobemide | 1140867920 | MAOI |
| manerix | 1140867922 | MAOI |
| maoi - tranylcypromine | 1140910820 | MAOI |
| tranylcypromine+trifluoperazine | 1140867944 | MAOI |
| maoi - phenelzine | 1140910704 | MAOI |
| maoi - isocarboxazid | 1140910504 | MAOI |
| marplan | 1140867858 | MAOI |
| parnate | 1140867916 | MAOI |
| selegiline | 1140879668 | MAOI |
| eldepryl | 1140872348 | MAOI |
| zelapar | 1141169666 | MAOI |
| mianserin | 1140879556 | NASSA |
| mirtazapine | 1141152732 | NASSA |
| zispin | 1141152736 | NASSA |
| bolvidon | 1140867806 | NASSA |
| venlafaxine | 1140916282 | SNRI |
| duloxetine | 1141200564 | SNRI |
| yentreve | 1141200570 | SNRI |
| cymbalta | 1141201834 | SNRI |
| edroanx | 1141151982 | SNRI |
| reboxetine | 1141151978 | SNRI |
| efexor | 1140916288 | SNRI |
| trazodone | 1140879634 | SARI |
| molipaxin | 1140882244 | SARI |
| nefazodone | 1140917460 | SARI |
| dutonin | 1140917466 | SARI |
| Bupropion | 1141176854 | NDRI |
| Methylphenidate | 1140917132 | NDRI |
| ritalin | 1140917138 | NDRI |
| concerta | 1141179874 | NDRI |

SSRIs= selective serotonergic reuptake inhibitors, TCA= tricyclic antidepressants, SNRI= serotonin-norepinephrine reuptake inhibitors, NASSA= noradrenergic and specific serotonergic antidepressant, MOAI= monoamine oxidase inhibitors, SARI= serotonin antagonists and reuptake inhibitors and NDRI= norepinephrine-dopamine reuptake inhibitors.

**Supplementary Fig 1A: The 1 sample Mendelian Randomization results for BMI in all individuals and in individuals not taking antidepressants. The odds ratios represent the change in binary mental health outcome per SD change in genetically ​determined BMI, 95% confidence interval in brackets.**

**Supplementary Fig 1B: The 1 sample Mendelian Randomization results for favourable adiposity in all individuals and in individuals not taking antidepressants. The odds ratios represent the change in binary mental health outcome per SD change in genetically determined favourable adiposity, 95% confidence interval in brackets.**

**Supplementary Fig 1C: The 1 sample Mendelian Randomization results for unfavourable adiposity in all individuals and in individuals not taking antidepressants. The odds ratios represent the change in binary mental health outcome per SD change in genetically determined unfavourable adiposity, 95% confidence interval in brackets.**

**Supplementary Fig 2A: The 1 sample Mendelian Randomization results for BMI in all individuals and in individuals not taking antidepressants. The betas represent the change in continuous mental health outcome per SD change in genetically determined BMI, 95% confidence interval in brackets.**

**Supplementary Fig 2B: The 1 sample Mendelian Randomization results for favourable adiposity in all individuals and in individuals not taking antidepressants. The betas represent the change in continuous mental health outcome per SD change in genetically determined favourable adiposity, 95% confidence interval in brackets.**

**Supplementary Fig 2C: The 1 sample Mendelian Randomization results for unfavourable adiposity in all individuals and in individuals not taking antidepressants. The betas represent the change in continuous mental health outcome per SD change in genetically ​determined unfavourable adiposity, 95% confidence interval in brackets.**
